# Development and Immunogenicity Assessment of a Multi-Epitope Antigen Against Zika Virus: An In Silico and In Vivo Approach

**DOI:** 10.3390/vaccines14010031

**Published:** 2025-12-26

**Authors:** Lígia Rosa Sales Leal, Matheus Gardini Amâncio Marques de Sena, Maria da Conceição Viana Invenção, Ingrid Andrêssa de Moura, André Luiz Santos de Jesus, Georon Ferreira de Sousa, Bárbara Rafaela da Silva Barros, Cristiane Moutinho Lagos de Melo, Lindomar José Pena, Francesca Paolini, Aldo Venuti, Anna Jéssica Duarte Silva, Antonio Carlos de Freitas

**Affiliations:** 1 Laboratory of Molecular Studies and Experimental Therapy—LEMTE, Department of Genetics, Federal University of Pernambuco, Recife 50670-901, Brazil; ligia.leal@ufpe.br (L.R.S.L.); matheus.gardini@ufpe.br (M.G.A.M.d.S.); maria.conceicao@ufpe.br (M.d.C.V.I.); ingrid.andressa@ufpe.br (I.A.d.M.); venutie4@yahoo.com (A.V.); anna.jessica@ufpe.br (A.J.D.S.); 2 Federal Institute of Education, Science and Technology of Mato Grosso–Lucas do Rio Verde Advanced Campus, Lucas do Rio Verde 78455-000, Brazil; andreluizsjesus@gmail.com; 3 Laboratory of Immunological and Antitumor Analysis, Keizo Asami Immunopathology Laboratory, Department of Antibiotics, Bioscience Center, Federal University of Pernambuco, Recife 50670-901, Brazil; georon.sousa@ufpe.br (G.F.d.S.); barbara.sbarros@ufpe.br (B.R.d.S.B.); cristiane.moutinho@ufpe.br (C.M.L.d.M.); 4 Department of Virology and Experimental Therapy, Aggeu Magalhães Institute, Oswaldo Cruz Foundation, Recife 50740-465, Brazil; lindomar.pena@fiocruz.br; 5 HPV-UNIT-UOSD Tumor Immunology and Immunotherapy, IRCCS Regina Elena National Cancer Institute, 00128 Rome, Italy; francesca.paolini@ifo.it

**Keywords:** Zika virus, DNA vaccine, multiepitopes, immunoinformatics, ssPGIP

## Abstract

**Background/Objectives:** The Zika virus (ZIKV) represents an ongoing threat to public health due to its neurological and congenital complications. Even after 10 years since the first major outbreak, correlated with an increase in congenital ZIKV syndrome, there is still no vaccine or treatment for this infection. Among the various existing platforms, DNA vaccines combined with the use of immunoinformatics tools allow for the efficient selection of immunogenic epitopes and immunostimulatory molecules with greater flexibility, in addition to being simple to manufacture and having a higher cost–benefit ratio in production. **Methods:** In this work, we conducted an integrated approach, combining in silico analyses and in vivo experimental validations, for the development of multi-epitope DNA vaccines against ZIKV. The computational analyses confirmed structural stability, adequate solubility, absence of toxicity, and immune induction potential for constructs based on epitopes from the Envelope (E) and NS1 proteins. Therefore, we evaluated DNA constructs containing the ENV + NS1 epitopes, both with and without fusion to the ssPGIP signal peptide, in BALB/c mice. **Results:** Both vaccines increased the population of CD4^+^ and CD8^+^ T lymphocytes, in addition to the production of IgG antibodies associated with the Th1 profile. The fusion with ssPGIP broadened the response, stimulating the release of Th1, Th2, and Th17 cytokines, as well as enhancing antibody formation. In contrast, its absence was associated with a slight increase in CD4^+^ and CD8^+^ T cells, accompanied by restricted cytokine production. **Conclusions:** These results indicate that epitope-targeted techniques offer a viable and safe method for inducing robust immune responses, demonstrating that combining immunoinformatics methods with early preclinical testing is an effective strategy for ZIKV vaccine development. Furthermore, although the present study focused on initial immunogenic characterization, future studies involving viral challenge in a suitable animal model will be essential to conclusively determine the protective efficacy of these vaccine candidates.

## 1. Introduction

The Zika virus, a member of the genus Flavivirus and the family Flaviviridae, is an enveloped, single-stranded, positive-sense RNA virus responsible for the 2015–2016 epidemic in the Americas [1]. It is associated with microcephaly in children, resulting in delayed or abnormal neuromotor development, a condition collectively known as Congenital Zika Syndrome (CZS), and is also linked to cases of Guillain-Barré Syndrome in adults [2,3]. Although there has been a reduction in reported incidence, the association between ZIKV and clinical manifestations remains a public health concern [4]. Furthermore, the correlation with potential co-infections and cross-immunity to other viruses in the family, such as dengue virus (DENV), yellow fever virus (YFV), and Japanese encephalitis virus (JEV), requires continued efforts to implement preventive measures [5,6,7].

Among these measures, notable studies in this field have reached preclinical and early clinical phase I and II stages [8,9,10] employing various strategies that include traditional inactivated virus platforms (NCT06334393; NCT03343626), adenovirus [11] and third-generation nucleic acid-based technologies ((mRNA) NCT03014089; NCT04064905; NCT02809443 (DNA)). Regardless of the platform, the E protein represents the most widely used antigen and the primary target for neutralizing antibodies [12]. This is due to its position as the outermost protein of the virion, serving as the principal component of viral envelope formation, and its critical role in membrane fusion during infection [13,14]. Different stages of viral infection progressively expose distinct regions of the E protein, rendering specific epitopes susceptible to neutralization at different developmental phases [12,15]. Beyond mediating humoral immunity, the E protein also acts as an activator of CD8^+^ T responses [16,17]. In contrast, other structural proteins, such as prM and capsid, exhibit lower exposure and limited accessibility. The capsid protein is associated with the genomic RNA and is located within the viral membrane and is therefore not directly accessible to antibodies [18]. The prM protein, in turn, is primarily exposed in immature viral particles, during early stages of the viral replication cycle, or as a result of inefficient virion assembly [19]. The immunogenicity of prM in vaccines has been shown to be mainly related to its role as an enhancer of immune responses against the E protein; however, when antibody responses directed against prM were evaluated in isolation, they exhibited low neutralizing capacity or activity restricted to the immature form of the virus, as well as an association with immunopathological reactions, such as antibody-dependent enhancement (ADE) [20,21]. NS1, a non-structural protein, on the other hand, induces protective humoral and cellular immune responses and circumvents the possibility of ADE development due to its absence from the surface of ZIKV virions. [22,23]. Furthermore, NS1 mediates immune evasion and is the only non-structural protein capable of being secreted into the extracellular environment, which enables it to be recognized by antibodies for the generation of a humoral response [24].

In this context, DNA vaccines offer significant advantages for exploiting these antigens, allowing the selection and rational design of specific immunogenic epitopes and immunostimulatory molecules with enormous flexibility. In addition, because its manufacture is relatively simple, it offers a cost-effective approach. Studies demonstrate that the immunogenicity of DNA vaccines can be optimized by incorporating genetic adjuvants, such as immunostimulant coding sequences and signal peptides, which enhance antigen presentation and the magnitude of the immune response [25,26]. Furthermore, bioinformatics strategies using reverse vaccinology allow the identification, selection, and optimization of suitable immunogenic epitopes for incorporation into a DNA multi-epitope vaccine [27,28]. This strategy enhances the favorable attributes of antigens, optimizes resource allocation, and minimizes the need to test previously considered less promising candidates [29], thus improving the translational efficiency between in silico predictions and experimental results.

Massa and collaborators [30], for example, demonstrated the potential of a signal peptide (ssPGIP) as an enhancer of the humoral response in therapeutic DNA vaccines against HPV-16. Similarly, the immunogenic potential of a multiepitope vaccine based on the NS1 and Envelope proteins of the Zika virus was recently evaluated in vitro and in vivo. This construct, composed of epitopes predicted in silico as possible targets for CD4, CD8, and B cells, proved to be a necessary component for an efficient response against the infection [31,32,33,34].

To this end, we utilized an integrated approach that included (i) in silico analyses to design and validate a multi-epitope construct (ENV + NS1) derived from the ZIKV E and NS1 proteins, focusing on its stability, safety, and immunogenic potential, and (ii) experimental validation in a murine model using DNA vaccines with the same construct, both with and without fusion to the ssPGIP signal peptide. The objective was to test the possibility of the multiepitope construct as an antigen for DNA vaccine, contributing to the development of a vaccine candidate against ZIKV.

## 2. Materials and Methods

### 2.1. Ethical Considerations

This study was approved by the Ethics Committee for the Use of Animals of the Instituto Aggeu Magalhães (IAM; Fundação Oswaldo Cruz–PE, Brazil), protocol number 110/2017. All animal experiments were carried out following the guidelines for biosafety and animal handling at the Animal House at the Instituto Aggeu Magalhães.

### 2.2. Study Design Overview

**Figure 1:**
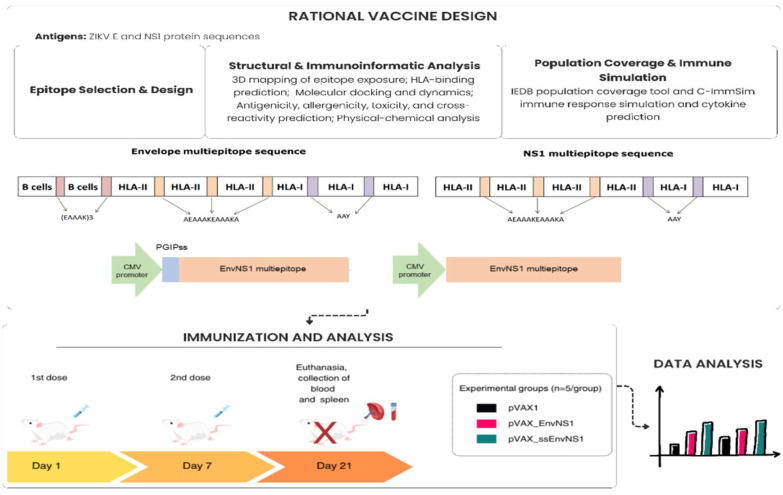
Rational design of plasmid construction and immunogenicity assessment scheme for the EnvNS1 multi-epitope DNA vaccine.

An integrated approach was employed, combining (i) the rational in silico design and analysis of multi-epitope vaccine constructs and (ii) subsequent experimental validation of immunogenicity in a murine model. The rational design is illustrated in Figure 1.

### 2.3. In Silico Rational Design and Analysis

#### 2.3.1. Epitope Selection and Construct Design

Eight epitopes from the E protein and six from NS1 were selected, previously predicted in earlier studies as capable of interacting with Major Histocompatibility Complex (MHC) class I and II molecules [35,36,37]. The epitopes were identified through pioneering studies utilizing predictive computational methods based on epitope-MHC binding assays. Furthermore, epitope conservation was evaluated across different geographic lineages, ranging from 30 to 54 lineages (Tables S1 and S2) in each study. Another selection criterion employed was in silico antigenicity and immunogenicity of epitopes for MHC class I.

The construct structure includes an *N*-terminal histidine signal sequence (8xHIS-tag) for immunodetection, a glycine linker—Gly (8)—to assist in proper protein folding, and concatenating epitopes directed toward activation of different lymphocyte types; specialized linkers were incorporated with distinct functions. Rigid specialized linkers (EAAAKEAAAKEAAAK, AEAAAKEAAAKA) were positioned between B cell epitopes and between epitopes for HTL, to prevent interference between sequences and confer appropriate conformation and bioactivity [38]. B cell epitopes were positioned near the *N*-terminal region to increase epitope exposure to the solvent and facilitate interaction with BCR [39]. In contrast to variation in presentation size, epitopes directed toward CTL possess greater length restriction for interaction in the binding groove [40]. To facilitate correct processing and prevent linker interference in the sequence, the AAY linker was utilized. The reduced linker size and capacity to act as a proteasomal cleavage site prevents the formation of junction epitopes, providing greater specificity of processing, thereby potentiating antigen presentation and induction of cytotoxic response [41].

#### 2.3.2. Structural Analysis and Epitope Exposure

The localization of the epitopes in the structure of the native proteins E (modeled via AlphaFold2 (v2023.11) [42] for incorporation of the transmembrane domain) and NS1 (obtained from the PDB: 5K6K) was determined using the ChimeraX 1.10.1 software [43]. This analysis aimed to verify the exposure of the epitopes in accessible surface regions, their presence in immunodominant domains, and critically, their potential overlap with binding sites of previously characterized neutralizing monoclonal antibodies. The monoclonal antibodies (mAbs) for E included in the analysis were MZ4, Z006, Z004, ZIKV-116, 7B3, ZK2B10, Z20, ZV-67, C8, ZIKV-117, A11, 1C11, Z3L1, and C10 [44,45,46,47,48,49,50,51,52,53], while the mAbs for NS1 included the highly neutralizing 4B8 and 3G2 and partially neutralizing 4F10, 2E11, and 14G5 [54].

#### 2.3.3. HLA Binding Prediction and Molecular Docking

The specific HLA alleles for each epitope were initially re-evaluated using the PROPRED and PROPRED1 tools. To refine affinity prediction, the IEDB Analysis Resource platform was employed [55]. Epitope–HLA complexes showing the highest concordance across the tools were selected for docking evaluation. The 3D structures of HLA alleles were retrieved from the RCSB PDB [56] (identifiers available in Figure S1), while epitope structures were modeled using PEPFOLD4 [57]. HLA structures were prepared in ChimeraX [43] for water and ion removal, hydrogen addition, and charge assignment. Molecular docking was performed using AutoDock Vina 1.1.2 via PyRx v0.8 software, to estimate binding affinity (binding free energy in kcal/mol), characterize molecular interactions and the positioning of the epitope within the HLA binding site [58,59]. Docking simulations were visualized and analyzed using Biovia Discovery Studio [60].

#### 2.3.4. Molecular Dynamics (MD) Simulations

To evaluate the temporal stability of the epitope–HLA complexes predicted by docking, Molecular Dynamics (MD) simulations were performed using the NAMD 3.0 software [61], utilizing the computational resources provided by the “National Center for High Performance Processing in São Paulo (CENAPAD-SP). The complexes were parameterized using the CHARMM-GUI server [62]. The CHARMM36m force field was used, and each system was solvated, neutralized with ions and subjected to an energy minimization protocol, heating to 310 K, with constant temperature, pressure and number of particles. Each simulation was performed in a period of 50 ns with analysis of 2500 frames. The trajectories were analyzed in VMD 2.0 to calculate key stability parameters: RMSD (Root Mean Square Deviation) of the ligand in the binding site, RMSF (Root Mean Square Fluctuation) of the complex (Epitope corresponds to the last 9–10 residues) and number of hydrogen bonds formed between the epitope and the HLA cleft [63]. A global integrated stability score was calculated for each complex from the normalization and combination of the mean plus the standard deviation of the results of the parameters, which were subsequently used to generate the heatmap. Graphs were generated using the latest stable versions available at the time of the study of the Python libraries NumPy and Pandas [64,65] for data extraction and Matplotlib and Seaborn [66,67] for visualization.

#### 2.3.5. Physicochemical Characterization

Parameters such as estimated half-life, isoelectric point (pI), stability index, aliphatic index, solubility, and average coefficient of hydropathicity (GRAVY) were calculated using ProtParam [68]. The solubility profile was predicted using the Protein-Sol tool against the *E. coli* soluble protein database [69].

#### 2.3.6. Prediction of Immunological and Safety Properties

Toxicity and allergenicity were predicted using machine learning-based binary classification tools such as ToxinPred [70] and AllerTop [71], respectively. To assess cross-reactivity, BLASTp (v2.17.0) alignments were performed against the human proteome (TaxID: 9606) and related flaviviruses: Dengue virus (TaxID: 12637), Yellow fever virus (TaxID: 11089), Japanese encephalitis virus (TaxID: 11072), and West Nile virus (TaxID: 11082). Antigenicity was predicted by the VaxiJen 2.0 tool [72] and cytokine induction was predicted for HLA class II epitopes using the IFNepitope [73], IL4Pred [74] and IL10Pred [75] servers.

#### 2.3.7. 3D Structure Modeling and Validation

The three-dimensional structure of the EnvNS1 construct was modeled using RoseTTaFold via the Robetta server [76]. The stereochemical quality was validated by Ramachandran plot analysis (Molprobity online web service) [77]. Stability was assessed using the CABS-flex 3.0 server, which evaluated RMSF in a dynamic environment to predict the flexibility of regions within the antigen structure [78].

#### 2.3.8. Population Coverage and Immune Response Simulation

The theoretical population coverage of the epitope set was calculated based on epitopes that showed intermediate/high interaction with the set of HLA tested in molecular dynamics. To perform this analysis, the IEDB Population Coverage Tool was used [79]. The immune response was simulated in silico using the C-ImmSim server [80], with a simulated immunization regimen of two doses seven days apart.

### 2.4. Experimental Validation In Vitro and In Vivo

#### 2.4.1. Viruses, Cells, and Reagents

The ZIKVPE243 isolate (GenBank reference number KX197192) was provided by Dr Lindomar Pena (Aggeu Magalhães Institute, FIOCRUZ, PE). Viral propagation was performed in VERO cells, as described by Baz, 2020 [81]. Viral titration was performed using the 6-well agarose overlay method [81]. HEK 293T cells used for transfection were cultured in DMEM medium (Sigma-Aldrich, St. Louis, MO, USA) supplemented with 10% fetal bovine serum (Gibco, Waltham, MA, USA) and 100 U/mL penicillin-streptomycin (Sigma-Aldrich).

#### 2.4.2. Vaccine Construction and Plasmid Production

Initially, the amplification of the sequence referring to the multiepitope construction was performed from the pCloneEnvNS1 vector and inserted into the pVAX1 vector (Invitrogen, Carlsbad, CA, USA). The plasmid pVAX-ss-L21–200-E7 * produced by Massa and collaborators [30], containing the ssPGIP signal sequence, was kindly provided by Dr Aldo Venuti (Regina Elena National Cancer Institute, Rome, Italy) and used as a template for amplification of ssPGIP. This sequence was fused to the *N*-terminal portion of the EnvNS1 sequence and inserted into the pVAX1 vector. Plasmid DNAs were used to transform *Escherichia coli* TOP10. All recombinant DNAs were digested by restriction enzymes and sequenced by the Sanger method to confirm the accuracy of the sequences. *E. coli* TOP10 cells containing pVAX1, pVAX_EnvNS1, or pVAX_ssEnvNS1 were cultivated under agitation at 37 °C in Luria–Bertani medium with kanamycin (50 μg/mL) for 16 h and extracted with the PureLink™ Expi Endotoxin-Free Maxi Plasmid Purification kit (Invitrogen).

#### 2.4.3. Mammalian Cell Transfection and Immunofluorescence

The expression of vaccine candidates was detected using immunofluorescence. For this, 1.10^5^ of HEK 293T cells were distributed per well in an 8-well Nunc^®^ Lab-Tek^®^ Chamber Slide System slide (Thermo Fisher Scientific, Waltham, MA, USA) and incubated until 50% confluence was established. The cells were then transiently transfected with the vaccine constructs (500 ng per well) and the pVAX1 empty vector with the transfection reagent Lipofectamine™ 3000 (Thermo Fisher Scientific) according to the manufacturer’s instructions and incubated for 24 h in an oven with 5% CO_2_ at 37 °C.

To perform fluorescence microscopy, cells grown on the multi-chamber slide were washed three times with ice-cold PBS pH 7.4, fixed for 20 min with 4% paraformaldehyde, permeabilized for 10 min with 0.1% Triton X-100 in PBS, and blocked with the blocking solution (1% BSA, PBS pH 7.6, 0.05% Tween 20 and 22.52 mg/mL glycine) for 1 h at room temperature (RT) under stirring. After blocking, the cells were incubated with a 1:3000 dilution of the Anti-6X His tag monoclonal antibody produced in mice (Sigma-Aldrich) in RT for 1 h under agitation. After removing the primary antibody, the cells were incubated with a 1:5000 dilution of the Anti-Mouse IgG antibody (whole molecule)–FITC produced in goats (Sigma-Aldrich) for 40 min in RT under agitation. Cell nuclei were counterstained with 0.5 µg/mL 4′,6′-diamino-2-phenyl-indole (DAPI) (Sigma-Aldrich). The slides were analyzed with the Leica DM2500 microscope, and the images were obtained with the CytoVision DM2500 program under 100× magnification.

#### 2.4.4. Experimental Groups and Immunization Regimen

BALB/c mice were raised and kept in the bioterium at the Instituto Aggeu Magalhães under specific pathogen-free conditions, and all animal experiments were carried out following the institutional guidelines for biosafety and animal handling. Groups composed of 5 female BALB/c mice aged 6–8 weeks were immunized by intramuscular injection into the left hind leg with 50 μg of pVAX_EnvNS1, pVAX_ssEnvNS1, or empty pVAX1 (control), in 30 μL of phosphate-buffered saline, twice with an interval of one week between immunizations. Immediately after each immunization, electroporation of the inoculation site was performed under the following conditions: 175 V/cm, 8 pulses, 20 ms (BTX Harvard-ECM^®^ 830, BTX, Holliston, MA, USA). Two weeks after the second vaccine dose, the animals were anesthetized (Xylazine Hydrochloride 10 mg/Kg and Ketamine 115 mg/Kg) and euthanized by cervical dislocation. The animals’ blood and spleen were collected for immunological, biochemical, and hematological tests.

#### 2.4.5. Immunogenicity Assessment

Splenocytes from immunized animals were treated with Ficoll-Paque PLUS 1077 g/mL (GE Healthcare Life Sciences, Marlborough, MA, USA) for the isolation of mononuclear cells. Isolated cells were distributed in 48-well plates with 10^6^ cells per well. Cells were restimulated with 10^5^ ZIKV-PE243 virus particles and cultured in RPMI 1640 medium (Sigma-Aldrich) supplemented with 10% fetal bovine serum (Gibco) for periods of 24, 48, and 72 h and analyzed for CD4^+^ and CD8^+^ T cell proliferation using anti-CD4-FITC and anti-CD8-PE antibodies (BD Bioscience, Franklin Lakes, NJ, USA), by flow cytometry reading 3000 events/analysis. The presence of cytokines (IL-2, -4, -6, -10, -17, IFN-γ, TNF) in plasma and splenocyte supernatants was evaluated using the CBA mouse TH1/TH2/TH17 cytokine kit (BD Bioscience). Plasma antibody isotypes (IgG1, IgG2a, IgG2b, IgG3, IgA, IgM, IgE) were quantified using a Mouse Immunoglobulin Isotyping CBA kit (BD Bioscience). Analyses were performed on a BD Accuri™ C6 Plus flow cytometer (BD Bioscience, Franklin Lakes, NJ, USA).

#### 2.4.6. Hematological and Biochemical Analyses

The blood samples were dispersed in microtubes containing EDTA K2 (Hemstab) and centrifuged at 500× *g* for 10 min to separate the plasma. Erythrogram, leukogram, platelet count, and biochemical analyses were performed as described by Silva et al., 2023 [32].

#### 2.4.7. Statistical Analysis

Graphs and statistical analyses were generated using GraphPad Prism version 9.0.0. Statistical differences between groups were assessed by analysis of variance with Ordinary one-way ANOVA, followed by Tukey’s post hoc tests. A *p*-value < 0.05 was considered statistically significant (* *p* < 0.05, ** *p* < 0.01, *** *p* < 0.001, **** *p* < 0.0001). To evaluate effect sizes, we observed R^2^ (R-squared) values that ranged from 0.6 to 0.9, indicating the relevance and practical significance of the data.

## 3. Results

### 3.1. Structural Localization and Neutralizing Antibody Overlap of Selected Epitopes

**Figure 2:**
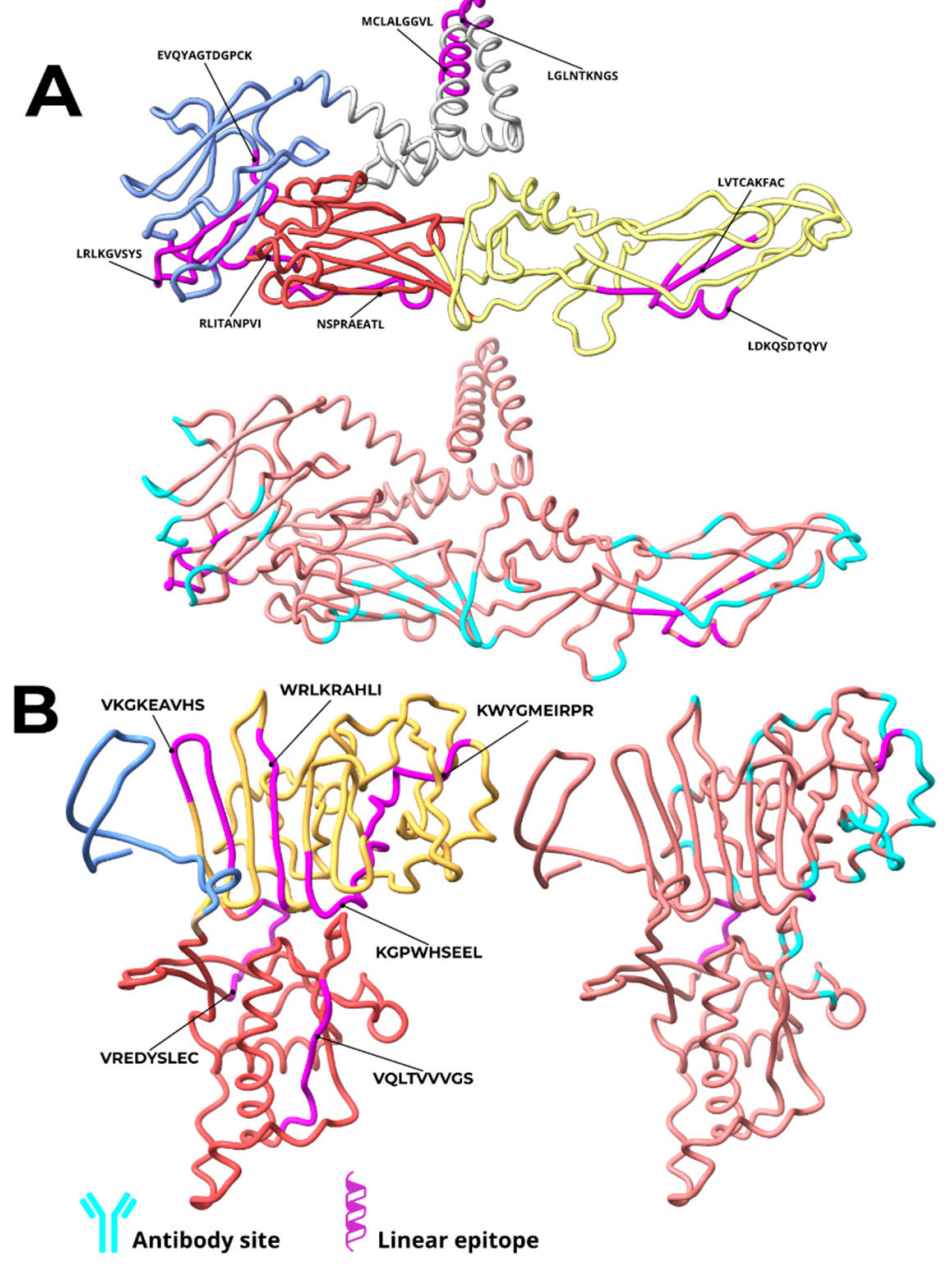
Location of epitopes in vaccine constructs derived from ZIKV E and NS1 proteins and critical regions for interaction with monoclonal antibodies. The epitopes of the constructs based on the E protein (**A**) are highlighted in pink and distributed across different domains: two in the transmembrane region (gray), three in domain III (blue), one in domain I (red), and two in domain II (yellow). For the NS1 protein (**B**), the epitopes (in pink) are located in two main domains: two in the β-ladder domain (yellow) and four in the Wing domain (red). The critical regions for interaction with mAbs are indicated in cyan.

Structural analysis of ZIKV E and NS1 proteins was conducted to determine the location of selected epitopes and their binding relationship to previously characterized mAb sites. The epitopes were predominantly located in exposed regions of the viral proteins, in domains considered immunologically relevant and capable of inducing neutralizing antibodies. For the E protein, six out of eight epitopes were identified in exposed structural domains: three in Domain III (DIII), one in Domain I (DI), and two in Domain II (DII). The remaining two epitopes were situated in the transmembrane region and are therefore unlikely to be accessible for antibody binding. No epitope was located in the fusion loop region of DII. Four exposed epitopes—RLKGVSYS and EVQYAGTDGPCK (DIII), LVTCAKFAC and LDKQSDTQYV (DII dimer interface)—were found to contain residues that directly overlap with the critical binding sites of highly neutralizing mAbs, including ZK2B10, ZIKV-117, and C8 (Table 1, Figure 2). Only three mAbs (1C11, Z3L1, and C10) analyzed did not target regions containing the selected epitopes.

For the NS1 protein, epitopes were dispersed across the wing domain (2 epitopes) and the β-ladder domain (4 epitopes). Three epitopes coincided with mAb interaction sites. The wing domain epitope VREDYSLEC contained five residues critical for binding to the highly neutralizing mAbs 3G2 and 4B8. Two epitopes in the β-ladder domain, KGPWHSEEL and CWYGMEIRPR, contained residues interacting with partially neutralizing mAbs (4F10, 2E11, and 14G5) (Table 2).

### 3.2. Molecular Docking Predicts Stable Epitope-HLA Binding

**Figure 3:**
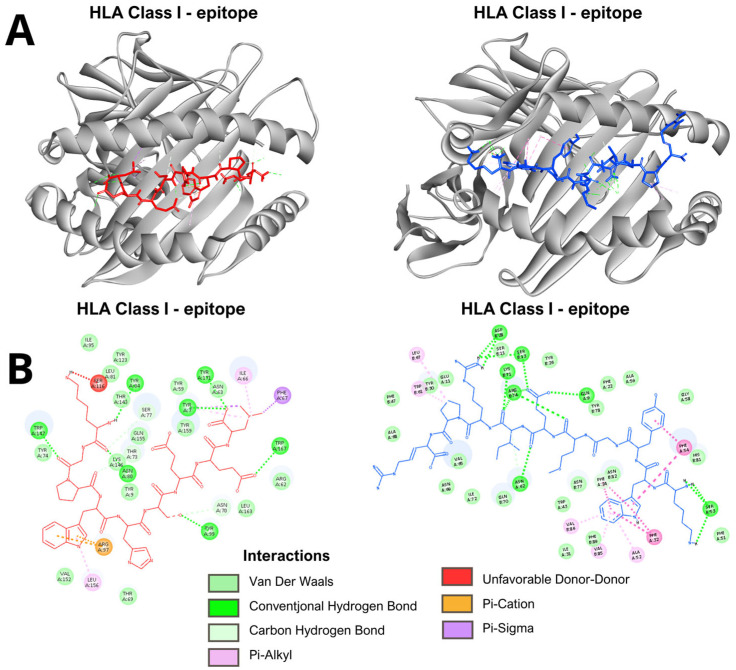
Representative results of the molecular docking analysis. (**A**). The models show the binding conformation of an epitope (red) within the peptide-binding groove of an HLA class I molecule (**left**) and an epitope (blue) within the groove of an HLA class II molecule (**right**). (**B**). Schematic representation of molecular interactions in epitope-HLA complexes. Detailed interactions for a representative HLA class I complex (**left**). Detailed interactions for a representative HLA class II complex (**right**). Key: Conventional Hydrogen Bond; Pi-Donor Hydrogen Bond; Pi-Sigma; Pi-Pi Stacked; Van der Waals; Unfavorable Donor-Donor. Directional non-covalent interactions are depicted as dotted lines based on geometric criteria.

In the analyses performed by molecular docking with the epitopes of the E and NS1 proteins, for both HLA class I and HLA class II, the conformations of the epitopes in the binding cleft, the binding affinity values of the complex and the interactions between the molecules were determined. All evaluated epitopes from both E and NS1 proteins successfully docked into the peptide-binding groove of their respective HLA class I and class II molecules, adopting conformations suitable for antigen presentation (Figure 3 and Figure S1). The binding affinities for HLA class I complexes varied from −6.5 to −9.8 kcal/mol, while those for HLA class II complexes ranged from −6.5 to −9.3 kcal/mol (Table 3 and Table 4). The examination of molecular interactions within the binding groove indicated a pattern of advantageous contacts essential for the stability of the complex. The primary interactions involved conventional hydrogen bonds, van der Waals forces, alkyl interactions and pi-based interactions, including pi-pi stacking, pi-sigma interactions, and pi-donor hydrogen bonds. Examples of these specific interactions for representative class I and class II complexes are detailed in Figure 3. Unfavorable donor-donor and acceptor-acceptor interactions were minimal across the complexes. The representative model and complete analysis are available in Figures S1 and S2 in supplementary material.

### 3.3. Binding Molecular Dynamics (MD) Simulations Reveal Epitope-HLA Complex Stability

**Figure 4:**
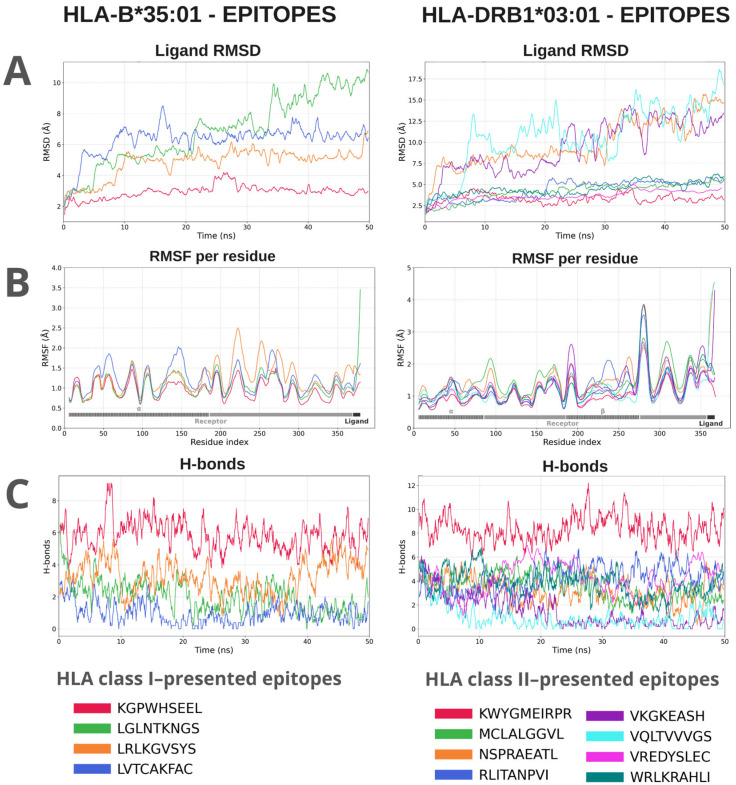
Molecular dynamics analyses of a representative from each group of class I and class II HLA complexes with their respective epitopes. The evaluated parameters were: RMSD (Å) of the ligand relative to the receptor, which demonstrates the displacement of the epitope within the binding groove along the trajectory. (**A**). RMSF (Å) shows the variation in the flexibility of the complex residues relative to the initial position, indicating fluctuations in the structure of the receptor and the ligand; the gray bar represents receptor residues, and the hatched region corresponds to the HLA interaction site. (**B**). The H-bonds indicate the variation in the number of hydrogen bonds formed between the ligand and the receptor throughout the simulation (**C**).

MD simulations were conducted to evaluate the stability of interactions between epitopes and HLA receptors. The epitopes exhibited variations in the interaction pattern with different HLA alleles, with distinctions observed in the stability parameters of the complexes. The global analysis of the 80 simulations showed a heterogeneous distribution of stability. Three groups were defined based on the integrated score obtained from the equilibrium phase of the simulations: Low stability (*N* = 48; score < 0.50): average RMSD of 4.77 Å, RMSF of 4.23 Å, and an average of 2.15 hydrogen bonds formed. Moderate stability (*N* = 26; score between 0.50 and 0.80): average RMSD of 3.43 Å, RMSF of 1.6 Å, and about 3.7 hydrogen bonds. High stability (*N* = 5; score > 0.80): lower RMSD values (2.77 Å) and RMSF (1.02 Å), in addition to an average of 6.33 hydrogen bonds throughout the simulation. The RMSD values of the entire set of complexes ranged between 1.1 and 6.7 Å (average of 3.90 Å), reflecting varying amplitudes of structural displacement in the simulated trajectories. The RMSF showed averages between 0.89 and 23.25 Å (overall average of 2.45 Å), indicating significant residual fluctuations in the evaluated regions. The average number of hydrogen bonds ranged from 0.45 to 13.1, with an average of 5.96, demonstrating differentiated persistence in the intermolecular interactions between epitopes and HLA alleles (Figure 4). The complete data plots of the molecular dynamics simulations can be found in Figures S3 and S4.

An integrated metric, constructed by normalizing and combining the scores, allowed for the generation of a heatmap (Figure S5), in which lighter colors indicate lower stability and reduced formation of hydrogen bonds, while darker shades of blue represent greater structural stability (better values of RMSD, RMSF, and number of H bonds). Complexes with higher integrated scores exhibited lower structural fluctuation and a higher number of hydrogen bonds, while lower scores indicated instability or complete dissociation of the immune receptor. Furthermore, the results showed that, except for epitopes targeting HLA-B40:01, HLA-B51:01, and HLA-B58:01, at least one of the evaluated epitopes exhibited an interaction classified as moderate with the total set of analyzed HLAs, suggesting a favorable potential for antigenic presentation by at least one HLA from the subset. Some epitopes demonstrated stability above the overall average. The complexes KGPWHSEEL:HLA-B*35:01 (0.84), LRLKGVSYS-HLA-B*08:01 (0.84), and LVTCAKFAC:HLA-A*68:01 (0.85) showed significant recognition and presentation capacity via MHC class I. The MHC class II complex KWYGMEIRPR:HLA-DRB1*03:01 (0.94) exhibited the highest score, indicating recognition affinity and potential T cell activation.

### 3.4. In Silico Characterization of the Multiepitope Construct

#### 3.4.1. Physicochemical Profile

The EnvNS1 construct was predicted to be soluble, stable, and hydrophilic. Key parameters included: instability index 30.39 (stable), aliphatic index 76.91 (thermostable), GRAVY −0.235 (hydrophilic), and solubility score 0.566 (soluble). Estimated half-life was >30 h in mammalian reticulocytes (in vitro), >20 h in yeast, and >10 h in *E. coli* (in vivo). These results are summarized in Table 5.

#### 3.4.2. Safety Profile and Immunogenicity

The multivalent antigen was classified as non-toxic and non-allergenic, with a VaxiJen antigenicity score of 0.4486 (probable antigen). Cytokine induction analysis revealed that seven of the eight HLA class II epitopes investigated demonstrated the capacity to induce IFN-γ, IL-4, and IL-10. Three NS1 epitopes, “KWYGMEIRPR”, “WRLKRAHLI”, and “VKGKEAHS”, showed potential to induce all three cytokines simultaneously. In contrast, the envelope epitope “NSPRAEATL” did not exhibit inductive capacity for any of the analyzed cytokines. The remaining epitopes displayed distinct induction profiles: “RLITANPVI” (E) induced IL-4 and IL-10; “VREDYSLEC” (NS1) induced IFN-γ and IL-10; “MCLALGGVL” (E) induced only IFN-γ; and “VQLTVVVGS” (NS1) induced IL-10.

In the cross-reactivity analysis, no significant similarity was found with the human proteome or with yellow fever virus proteins, as presented in Table 6. Throughout the entire protein, only a region corresponding to the epitope “KWYGMEIRP” presented significant similarity with an epitope of the NS1 protein of dengue virus. The overlapping region of the alignment, corresponding to 4% of the query cover of the multi-epitope construct, consisted of 81.82% identity residues, which refers to identical residues between the aligned sequences. Additionally, low correspondence was observed with West Nile virus (40.54% identity, 14% query coverage and 40.54% for E) and with Japanese encephalitis virus (29.89% identity, 25% query coverage).

#### 3.4.3. Population Coverage

The epitope set showed broad theoretical population coverage: 87.28% worldwide, 75.83% for Brazil, and 60.59% for South America. The individual coverage of the epitope set for each geographical context, including global worldwide, national Brazil, and regional South America, can be seen in Table 7.

#### 3.4.4. Immune Response Simulation

**Figure 5:**
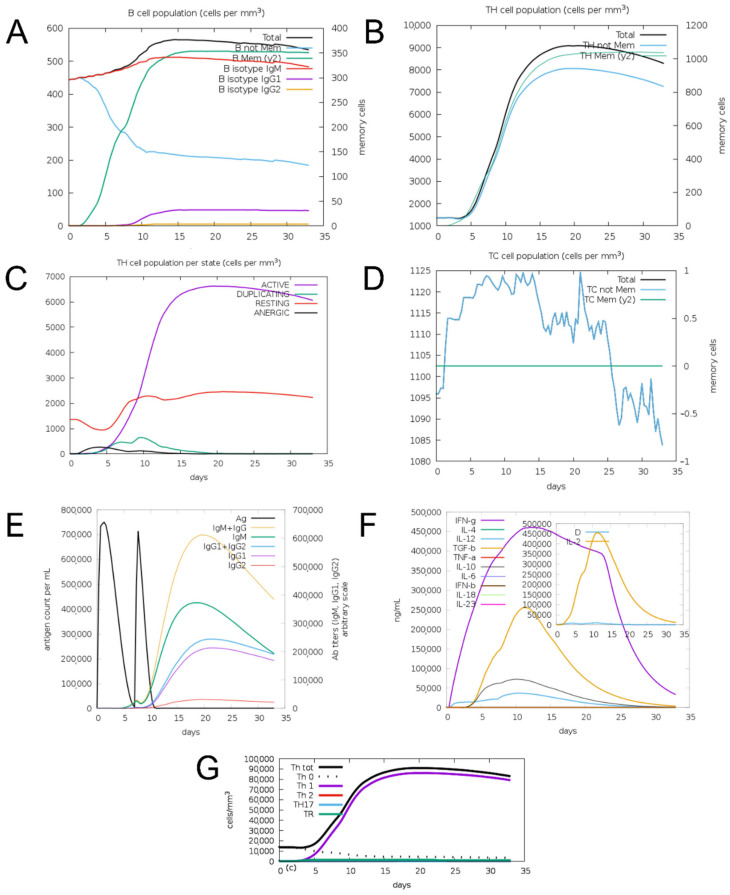
Results of prediction of immune response induction for the EnvNS1 construct. (**A**) B cell population; (**B**) Helper T cell population; (**C**) Helper T cell response profile; (**D**) Cytotoxic T cell population; (**E**) Induction of antibody production; (**F**) Induction of cytokine production; (**G**) T helper cell response profile.

C-ImmSim simulation predicted a Th1-type immune response with production of IFN-γ, TGF-β, IL-10, IL-12, and IL-2. The construct stimulated B-cell and T-helper cell and T-cytotoxic cell populations and induced production of IgM, IgG1, and IgG2 antibodies (Figure 5).

#### 3.4.5. Structural Characterization

**Figure 6:**
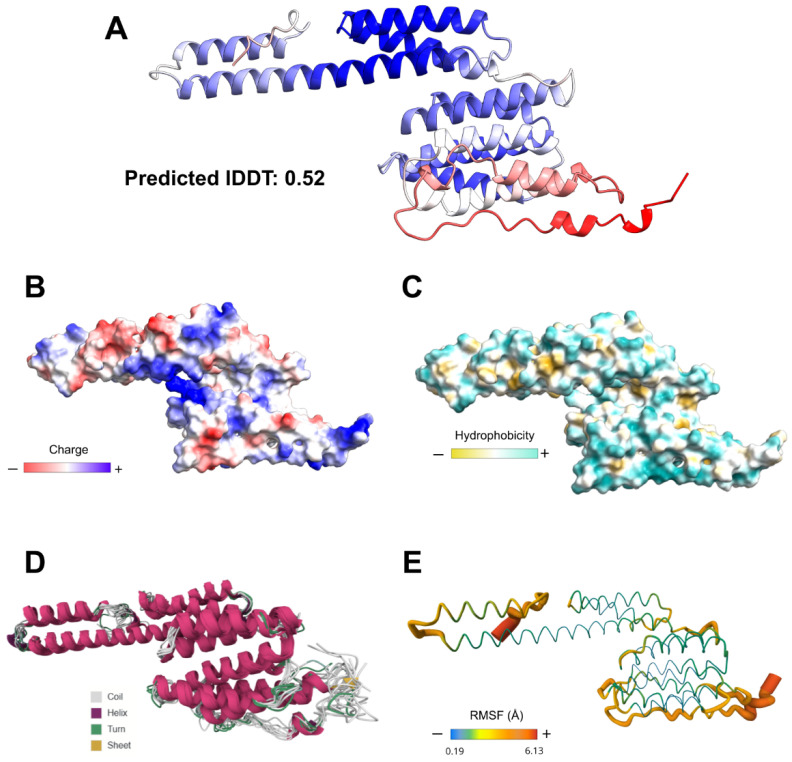
3D modeling and structural validation of EnvNS1. (**A**) RoseTTaFold-predicted structure with confidence coloring (blue: high, red: low). Surface properties relative to electrostatic charge distribution (**B**), hydrophobicity (**C**), flexibility (RMSF) and thermostability (**D**,**E**).

The generated 3D model presented a general lDDT score of 0.52. Stereochemical quality assessment of the modeling yielded a score of 96.8% of residues in favored regions on the Ramachandran plot and 0.3% in disallowed regions (Figure S6). Surface analysis indicated a hydrophilic character and balanced charge distribution. RMSF analysis of the three-dimensional structure suggested that the EnvNS1 construct has low propensity for structural deformations and low flexibility, indicating minimal to mild susceptibility to temperature variations (Figure 6). The resulting structure also enabled the assessment of epitope and linker distribution along the predicted three-dimensional antigen (Figure S7).

### 3.5. Construct Designs, Cloning in the pVAX1 Vector, and Immunofluorescence

**Figure 7:**
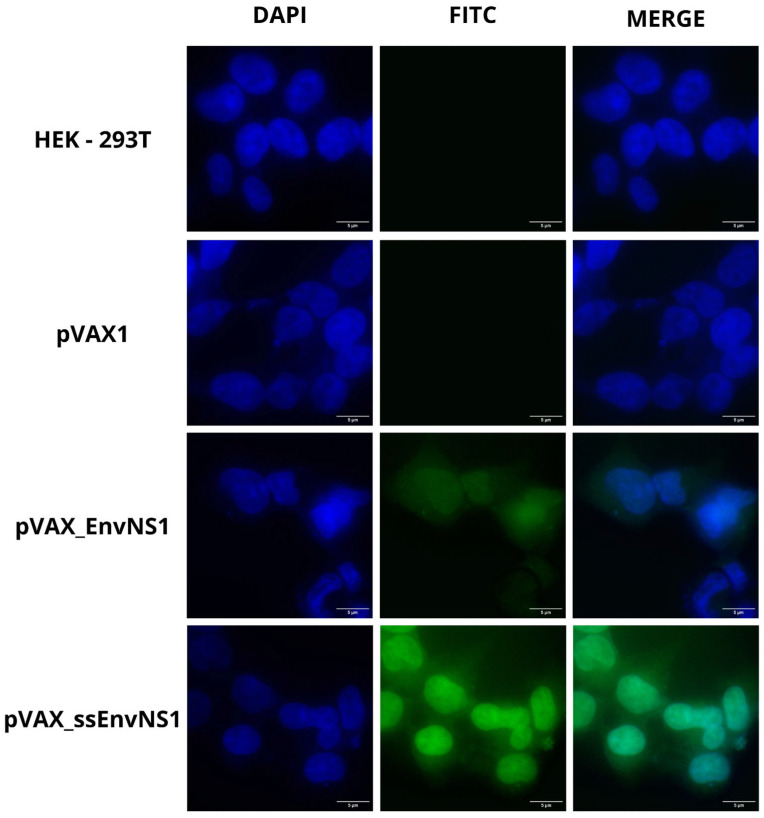
Representative fluorescence microscopy (FITC) images in cells transfected with pVAX1, pVAX_EnvNS1, and pVAXssEnvNS1. Non-transfected HEK 293T cells were used as an autofluorescence control. Image capture was performed at 100× magnification.

The EnvNS1 multiepitope sequence and its fusion version with the ssPGIP signal peptide were cloned into the pVAX1 vector. The epitopes were arranged into groups of immunological targets and separated with the linker sequences EAAAKE-AAAKEAAAK (between B cell epitopes), AEAAAKEAAAK (between CD4 T epitopes) and AAY (between CD8 T epitopes). After cloning the EnvNS1 and ssEnvNS1 sequences in pVAX1, enzymatic digestion was performed with *Nhe*I and *Xho*I enzymes to confirm the recombinant clones referring to the vaccine candidates (Figure S8). Immunofluorescence analysis of HEK 293T cells transfected with pVAX_EnvNS1 and pVAX_ssEnvNS1 was performed to confirm the expression of proteins encoded by vaccine candidates (Figure 7). For both DNA constructions, the presence of fluorescence in the cytoplasm and intracellular membranes was observed, with increased fluorescence associated with the expression of ssEnvNS1.

### 3.6. Analysis of Th1, Th2, and Th17 Cytokines Found in Blood and Produced by Virus-Stimulated Splenocytes

**Figure 8:**
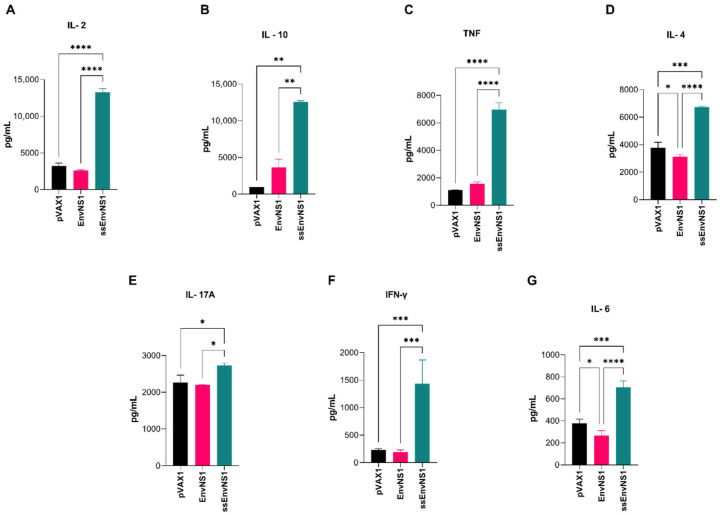
Profile of cytokines measured in the serum of vaccinated mice. The collection was performed 14 days after the last dose. (**A**) IL-2, (**B**) IL-10, (**C**) TNF, (**D**) IL-4, (**E**) IL-17A, (**F**) IFN-γ, (**G**) IL-6. Cytokine values were measured in pg/mL. Asterisks represent statistical significance (* *p* < 0.05, ** *p* < 0.01, *** *p* < 0.001, **** *p* < 0.0001).

Groups composed of five BALB/c mice were intramuscularly immunized with either one of the vaccine candidates or the empty vector pVAX1 and boosted with the same choice after seven days. After 14 days of the second dose, blood and spleen were collected from the immunized animals and the pVAX1 control group (Figure 1). The plasma obtained from collected blood samples was analyzed for circulating cytokines. No significant detection of cytokines was observed in animals from the pVAX_EnvNS1 group when compared to the pVAX1 group. However, for the pVAX_ssEnvNS1 analyses, a significant production of IL-2, IL-4, IL-6, IFN-γ, TNF, IL-17A, and IL-10 was observed (Figure 8A–G) when compared to both the pVAX1 and EnvNS1 groups, with particular emphasis on the levels of IL-2, TNF, IL-4, IFN-γ, and IL-6. These data indicate a mixed Th1/Th2/Th17 response profile in the group immunized by pVAX_ssEnvNS1.

**Figure 9:**
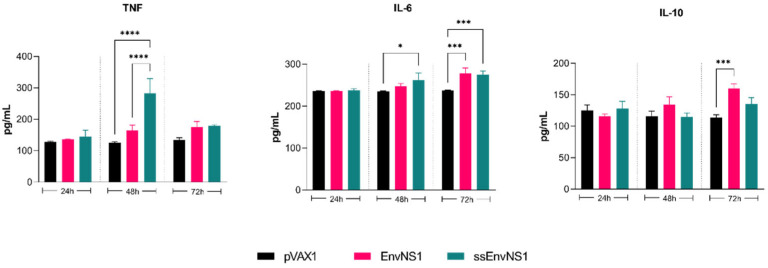
Profile of TNF, IL-6, and IL-10 cytokine levels produced by ZIKV-stimulated splenocytes for periods of 24, 48, and 72 h of culture. Cytokine values were measured in pg/mL. Asterisks represent statistical significance (* *p* < 0.05, *** *p* < 0.001, **** *p* < 0.0001).

To assess a possible memory immune response, cytokines secreted by splenocytes of immunized mice stimulated in vitro with ZIKV were measured (Figure 9). The splenocytes from the pVAX_ssEnvNS1 group exhibited a pro-inflammatory response, showing a peak in TNF release and an increase in IL-6 levels after 48 and 72 h of stimulation, respectively. Splenocytes from the pVAX_EnvNS1 group produced similar levels of IL-6 as the ssEnvNS1 group at 72 h and significant IL-10 secretion on the third day of stimulation. No significant levels of IL-4, IL-2, IFN-γ, or IL-17A were observed throughout the established stimulation periods. These profiles indicate a similar response trend among the groups, with a Th1 response trend observed in both the pVAX_ssEnvNS1 group and the pVAX_EnvNS1 group.

### 3.7. Detection of CD4^+^ and CD8^+^ T Cells After Two Vaccine Doses and Proliferation of Isolated Splenic Lymphocytes in Response to Viral Stimulation

**Figure 10:**
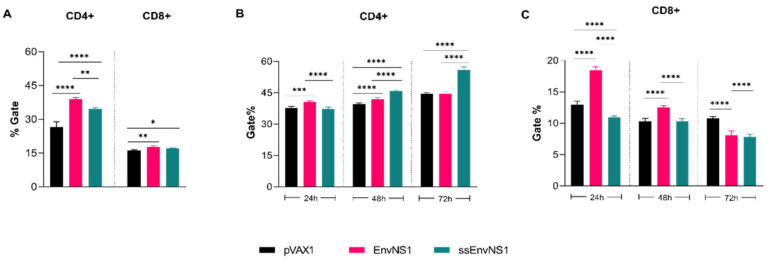
Analyzes of subpopulations of lymphocytes present in the blood of mice after immunization and of lymphocytes activated after viral stimulation. Sample collection was performed 14 days after the last dose. (**A**) CD4^+^ and CD8^+^ blood lymphocytes. (**B**) Spleen-derived CD4^+^ T cells after ZIKV stimulation. (**C**) Spleen-derived CD8^+^ T cells after ZIKV stimulation. Asterisks represent statistical significance (* *p* < 0.05, ** *p* < 0.01, *** *p* < 0.001, **** *p* < 0.0001).

Analysis of CD4^+^ and CD8^+^ T lymphocyte percentages after immunization was conducted using flow cytometry. The gate strategy for immunophenotyping analyses is available in Figure S9. A significant increase in the number of CD4^+^ cells was observed in the blood samples of immunized animals, with a higher percentage observed in the group immunized with pVAX_EnvNS1 (Figure 10A). As for the population of CD8^+^ T cells, a slight proliferation was observed in the groups immunized with pVAX_EnvNS1 and pVAX_ssEnvNS1 (Figure 10A). The activation of CD4^+^ and CD8^+^ T lymphocytes in response to the ZIKV stimulus was evaluated over 24 h, 48 h, and 72 h of culture of splenocytes from immunized mice. When analyzing the population of CD4^+^ T cells (Figure 10B) at the 24-h mark, a significant difference was observed between pVAX_EnvNS1 and pVAX_ssEnvNS1. However, there was an increase in the observed percentage of pVAX_ssEnvNS1 at the 48-h culture. In contrast to the observations at 24 h and 48 h, there was a significant increase in the response to pVAX_ssEnvNS1 at 72 h. Regarding the evaluations for CD8^+^ lymphocytes (Figure 10C), a peak response for pVAX_EnvNS1 was observed in the 24-h culture, with strong significance. However, there was a decrease in the level observed for pVAX_EnvNS1 in the 48-h culture, although it remained statistically significant compared to the other groups. For both immunized groups, there was a reduction in detection at 72 h.

### 3.8. Detection of Antibodies in the Plasma of Immunized Mice

**Figure 11:**
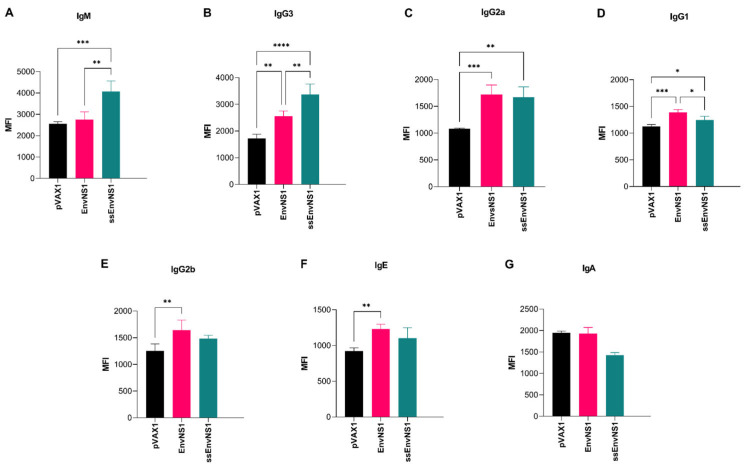
Antibodies produced in response to immunization in the sera of vaccinated mice. Sample collection was performed 14 days after the last dose. (**A**) IgM, (**B**) IgG3, (**C**) IgG2a, (**D**) IgG1, (**E**) IgG2b, (**F**) IgE, (**G**) IgA. Cytokine values were measured in MFI. Asterisks represent statistical significance (* *p* < 0.05, ** *p* < 0.01, *** *p* < 0.001, **** *p* < 0.0001).

The humoral response resulting from immunization was assessed by isotyping antibodies present in the plasma of immunized mice. A robust induction of the innate immune response, as represented by the level of IgM, was observed in animals immunized with pVAX_ssEnvNS1 (Figure 11). Additionally, significant levels of IgG3, IgG2a, and IgG1 were observed in this group 14 days after the booster dose (Figure 11). In contrast to the response seen with pVAX_ssEnvNS1, immunization with pVAX_EnvNS1 did not result in significant production of IgM. Instead, it induced the production of IgG3, IgG2a, IgG1, IgG2b, and IgE (Figure 11). Neither of the immunized groups exhibited IgA production (Figure 11). By analyzing the ratios calculated between the IgG2a, IgG2b, and IgG1 subtypes, trends in the profile of the immune response associated with immunization, either Th1 (IgG2a) or Th2 (IgG1 and IgG2b), can be identified [82]. For this purpose, the ratios (IgG1/IgG2a) and (IgG2b/IgG2a) were calculated, where values less than 1 were considered indicative of a Th1 response (Table 8). Both vaccines exhibited values lower than 1, indicating the induction of a Th1-skewed response, with slightly lower values observed for pVAX_ssEnvNS1.

### 3.9. In Life Assessments, Hematological and Biochemical Analysis

The weight, apparent activity level of the animals, and the sites of inoculation and electroporation were observed to detect any changes during the vaccination schedule. No significant behavioral alterations, such as aggressiveness or lethargy, were observed throughout the experimental period. Moreover, there were no signs of damage at the inoculation and electroporation sites. All animals were weighed before the immunization events (day 1 and day 7) and upon euthanasia on the 21st day. No significant changes in weight were noted. The averages per group for each weighing are listed in Table S3 of the Supplementary Materials. To assess whether the immunization process caused any clinical alterations in the vaccinated mice, hematological and biochemical analyses were performed on blood collected at the end of the vaccination schedule. Regarding the hematological parameters, no significant changes were observed, and the measured values were similar across all groups (Table S4). Similarly, no significant changes were found when analyzing the biochemical parameters assessed in all groups (Table S5).

## 4. Discussion

This study presents a rational strategy for vaccine development that integrates computational prediction with initial in vivo validation. Our approach began with the in silico analysis of the multi-epitope construct EnvNS1, which provided a deeper understanding of its structural and immunological properties before proceeding to in vivo tests, which demonstrated measurable immunogenicity but revealed opportunities for improvement. Signal peptides such as tPA, gp67, and the IgE signal sequence, the latter present in the first DNA vaccine for human use against SARS-CoV-2 infection [83,84], are commonly employed for increased antigen expression and immunogenicity [85]. Additionally, some studies have demonstrated that using heterologous signal peptides from various species can be effective in increasing protein translation efficiency and, as a result, improving protein production and bioavailability [86,87,88]. This was also observed in the application of plant peptide signal ssPGIP, which was shown to successfully improve processing, antigen presentation, immunogenicity of two DNA vaccines against other pathogens [30,89]. These preliminary observations motivated the incorporation of the ssPGIP signal peptide in this study.

The in silico analyses revealed several favorable features supporting further development. The physicochemical analysis, for instance, predicted a solubility of 0.566, a hydrophilic nature (GRAVY −0.235), and an instability index of 30.39—considered positive results for recombinant protein expression and vaccine formulation. The stability assessment indicated thermostability due to the aliphatic amino acid composition of the sequence and low structural flexibility in the three-dimensional model. The stability of recombinant antigen constructs directly influences their interaction with the immune system and subsequent processing. Stable structures tend to stimulate the immune system for a longer period and generate more durable responses, whereas overly stable antigens may hinder effective presentation by reducing intracellular proteolytic processing within APCs [90].

Safety assessments indicated a favorable profile, with no evidence of toxicity or allergenicity. Furthermore, the cross-reactivity analysis of the construct identified similarities between the sequence of the epitope “KGPWHSEEL” from NS1 and the DENV4 sequence. The potential risk of cross-reactive epitopes with DENV is mainly related to the development of ADE; however, the identified epitope originates from the NS1 protein, which is not related to the development of this phenomenon, as it is absent in flavivirus virions. Anti-NS1 antibodies have been shown in some cases to be protective against DENV and ZIKV infections [91,92]. The NS1 protein has a greater presence of cross-reactions against the human proteome, being identified in events of hepatic lesions, endothelial damage, and autoimmune neurological alterations such as Guillain-Barré Syndrome [91,93,94]. The cross-reactivity result against the human proteome indicated absence of substantial similarity with human proteins, indicating reduced capacity for autoantibody induction. Regarding cross-reactivity against E epitopes, similarity was found against some clinically relevant flaviviruses, such as WNV and JEV, although with low identity (>50%) in the regions considered significant, which indicates low possibility of cross-reactive responses against epitopes or conserved epitope fragments between species. The low similarity increases the specificity of the vaccine candidate, reducing the probability of inducing cross-reactive antibodies and therefore preventing undesirable reactions, such as those associated with antibody-dependent enhancement (ADE) of infection mediated by non-neutralizing anti-E antibodies that favor DENV infection [95].

Structural mapping identified regions corresponding to binding sites of highly neutralizing monoclonal antibodies, suggesting that induction of similar antibodies targeting these regions through the identified linear epitopes may trigger comparable immune responses. Four linear epitopes derived from the envelope protein and three from NS1 were identified as candidates capable of eliciting antibody production toward these regions. The envelope epitopes “EVQYAGTDGPCK”, “LVTCAKFAC” and the NS1 epitope “VREDYSLEC” showed particularly high coverage, indicating promising potential for neutralizing antibody production. Additionally, molecular docking simulations revealed binding of the epitopes to multiple HLA alleles, with affinities ranging from −6.5 to −9.8 kcal/mol. These values are comparable to those reported by Prasasty et al. [96], who performed molecular docking of ZIKV epitopes identified as HLA ligands.

Molecular dynamics (MD) simulations revealed greater variation than observed in the static molecular docking results when the complexes were assessed over time under conditions closer to a physiological environment. This analysis identified complexes—such as those formed by epitopes LVTCAKFAC and KGPWHSEEL with their respective Class I alleles, and WRLKRAHLI, RLITANPVI, and KWYGMEIRPR with their corresponding Class II alleles—that consistently stood out as the most stable. These highly stable complexes likely possess strong potential for antigen presentation to T cells. The RMSD and RMSF values obtained for this highly stable group are consistent with stable epitope interactions within the HLA binding groove, as reported by Da Costa et al. [97]. Moreover, within the intermediate-interaction group, which included most epitopes, broad coverage across different HLA alleles was observed, suggesting presentation potential for diverse population groups. Low dissociation tendency and maintenance of a compatible binding conformation over time are essential properties for efficient and sustained antigen presentation, capable of eliciting effective T-cell responses [98]. If efficiently presented across the evaluated HLA set, the construct may achieve broad population coverage and potential efficacy in different genetic contexts (87.28 globally and 75.83% for Brazil).

The immunological simulations correlated well with experimental data, showing a Th1-biased response, also observed in experimental results for EnvNS1 and ssEnvNS1. Furthermore, the in silico response was characterized by CD4^+^ and CD8^+^ cell activation and IgG1 and IgG2 antibody production. Consistent with this in silico immune response, cytokine induction predictions indicated that the epitopes within the construct could promote cytokine production. The predicted in silico immune response suggested cytokine induction potential, primarily involving IFN-γ and IL-10, with NS1 being the main contributor.

On the other hand, from the perspective of the results obtained from in vivo experiments, the analysis of systemic cytokines present in the plasma of the animals 14 days after the booster dose indicates that immunization with pVAX_EnvNS1 did not induce a lasting production of any of the evaluated cytokines. This directly contrasts with what was observed for the group immunized with pVAX_ssEnvNS1, which led to an increase in the production of all the evaluated cytokines. Despite inducing cellular and humoral responses, as reported in clinical trials with humans, DNA vaccines are poorly immunogenic, which is one of the challenges for their use [99]. Several events seem to be involved, including the rapid degradation of the DNA molecule, the need for its relocation to the cell nucleus for transcription, and the final localization of the encoded protein [100,101]. The data obtained from immunofluorescence analyses indicate the cytoplasmic localization of the proteins encoded by the EnvNS1 sequence. It is interesting to observe, however, the higher fluorescence intensity captured by the cells transfected with ssEnvNS1. Thus, it is possible that the fusion of ssPGIP with the multi-epitope sequence directed the nascent protein to the ER lumen, allowing for more efficient translational and post-translational processing, thereby enabling a higher level of synthesized protein [102].

The analysis of the cytokines produced in the plasma in response to immunization with pVAX_ssEnvNS1 showed significant production of all the evaluated molecules, indicating a multifunctional profile of T cell production. This finding is particularly interesting as a polyfunctional immune activation has been demonstrated during both acute and recovery phases of ZIKV infection [103,104,105]. Immunization with pVAX_EnvNS1 induced a discrete production of IL-10 and TNF. However, these results are not significant when compared to the pVAX1 control group. The splenocytes from immunized animals were stimulated in vitro with ZIKV to detect the cytokines produced in response. For the pVAX_ssEnvNS1 group, there was production of TNF and IL-6, while in the pVAX_EnvNS1 group, there was production of IL-6 and IL-10. Several studies demonstrated the role of Th1-mediated response in active protection against ZIKV infection [17,106,107,108] and even in memory responses years after infection in humans [109]. In this regard, though slightly differentiated, the evaluation of the produced cytokines indicates a distinct trend towards a Th1 profile for both vaccinal constructs. Elevated levels of cytokines derived from various T cell subpopulations are observed during acute Zika virus infection, with a delicate balance between pro- and anti-inflammatory responses present in the initial response to viral infection. Cytokines associated with Th1, Th2, Th9, and Th17 profiles, were the most reported [106,107,108]. While in the convalescent phase, the production of TNF, IFN-γ, IL-1β, IL-6, IL-8, IL-10, and IL-13 is observed [106,107].

Research involving infected humans supports the protective role of CD4^+^ and CD8^+^ effector T cells against dengue virus infection [110,111]. Similarly, the induction of a strong T cell response is likely necessary for the development of effective vaccines against the Zika virus, but much still needs to be clarified regarding the defined roles of the cell subtypes involved in the protective immune response against Zika virus infection [112,113]. As part of the characterization of the immunogenicity profile of the DNA vaccines in this study, analyses of the CD4^+^ and CD8^+^ T cell subpopulations were conducted by evaluating the presence of these circulating subpopulations after the immunization. Among the subpopulations analyzed from the blood, the highest proliferation was observed for CD4^+^ T cells in both immunized groups, with a higher percentage of these cells observed in the pVAX_EnvNS1 immunized group.

The splenocytes stimulated in vitro demonstrated a distinct profile, with the proliferation of lymphocytes derived from the pVAX_EnvNS1 group occurring only in the first 24 h of culture. On the other hand, the splenocytes from the pVAX_ssEnvNS1 group exhibited a progressive increase in proliferation, lasting up to 72 h. CD4^+^ T cells play a fundamental role in the generation and long-term maintenance of the antibody-mediated response; thus, activation through vaccination is important [114,115]. Animal studies have shown that Zika virus infection stimulates the growth of CD4^+^ follicular T cells (Tfh) and regulatory T cells, which are necessary for producing neutralizing antibodies against the infection [17,116].

Hassert et al. [117] evaluated the effects of CD4^+^ T cell depletion in Ifnar1-/- mice infected with the Zika virus and observed severe neurological damage and high viral load in the central nervous system of infected animals in the absence of CD4^+^ T cells. Furthermore, they demonstrated that the transfer of CD4^+^ T cells from immunized mice was sufficient to protect against lethal challenge in mice susceptible to Zika virus infection [117]. Immunization with pVAX_ssEnvNS1 and pVAX_EnvNS1 induced a moderate systemic proliferation of CD8^+^ T cells, as analyzed at the end of the experimental period. On the other hand, during the in vitro stimulation of splenocytes from immunized mice, stimulation was observed only in cells derived from the pVAX_EnvNS1 group, with proliferations detected between 24 and 48 h.

It is possible that the discrete activation of CD8^+^ T cells occurred due to the lower processing of the selected epitopes, which in turn are also present in a smaller number of targets in the multi-epitope construct (HLA-A: 3/14; HLA-A and DR: 2/14). Although evidence indicates that the CD4^+^ T cell-mediated response is induced by epitopes present throughout the ZIKV genome, the CD8^+^ T cell-mediated response is strongly directed toward epitopes present in the virus’s non-structural proteins [118]. However, one of the epitopes present in the multiepitope construct, NS1262-275, was characterized as immunodominant in the response to ZIKV infection with potential cytotoxic activity [119]. In any case, it is important to consider that the CD8^+^ T cell epitopes selected for this research are mainly derived from the E protein, which may not favor the response mediated by this cell subpopulation.

To evaluate the humoral response elicited by DNA vaccines, an antibody typing was conducted with plasma samples obtained from mice following the completion of a 21-day immunization schedule. Significant levels of IgM were detected in the group immunized with pVAX_ssEnvNS1 14 days after the last vaccine dose. In natural ZIKV infection, IgM levels often remain positive from the fourth day post-symptom onset for up to 12 weeks, and occasionally longer [120]. In individuals previously infected with the dengue virus, IgM antibodies specific to ZIKV showed little cross-reactivity and were able to induce a neutralizing response [121].

Both vaccines showed a similar stimulation for the production of IgG3, IgG2a, IgG1, IgG2b, and IgE antibodies. The results corroborate the function of Th1-like Tfh cells in the maturation of the humoral response to ZIKV, recognized as vital for the neutralizing response and the class switching of IgG2a antibodies in BALB/c mice [116]. In addition, the data obtained suggest a Th1-polarized humoral stimulation for pVAX_ssEnvNS1 and pVAX_EnvNS1. It is important to note that these interpretations may be limited by the chosen study model [122] and cannot be directly related to the course of the immune response in infected humans.

## 5. Conclusions

The rational strategy that integrates an in silico design with the incorporation of the ssPGIP signal peptide was decisive for the success of the pVAX_ssEnvNS1 candidate, which presents itself as a promising DNA vaccine with an immunogenic profile and favorable characteristics for future development as a prophylactic strategy against ZIKV. Despite the results obtained, this study has limitations, such as the use of electroporation to deliver DNA vaccines, which would prove to be difficult to use in large immunization programs, as opposed to the use of microneedles for enhanced delivery. Other limitations include the use of immunocompetent BALB/c mice without exposure to the viral challenge, the absence of evaluation of human-specific MHC allele presentation to induce T responses and the lack of data on the neutralizing capacity of the antibodies produced in response to the vaccines evaluated in this research. In this sense, additional analyses of the neutralizing capacity of the antibodies induced by immunization, as well as the evaluation of protection induction through immune challenge, will be necessary to determine the viability of using these immunizers in prophylactic vaccine strategies against the Zika virus.

## Figures and Tables

**Table 1:** Epitopes from ZIKV E protein overlap with critical binding residues of highly neutralizing monoclonal antibodies (mAbs). mAbs are listed in the first column. Residues within epitopes that are critical for mAb interaction are shown in bold. The final column indicates the protein domain of each epitope.

	LRLKGVSYS	EVQYAGTDGPCK	LVTCAKFAC	LDKQSDTQYV	DOMAIN
MZ4 [46]	LRLK**G**VS**Y**S				**DI/DIII linker**
Z006 [47]	LRLKGVSY**S**				**DIII**
Z004 [48]	LRLK**G**VS**Y**S	EVQYAG**T**DGPCK			**DI/DIII linker**
ZIKV-116 [51]	LRLKGVSY**S**	EVQYAGT**D**GPCK			**DIII**
7B3 [44]		EVQYAG**T**D**G**PCK			**DIII**
ZK2B10 [52]		EV**Q**Y**A**GTDGPCK			**DIII**
ZK-67 [45]		EVQY**A**G**T**DGPCK			**DIII**
C8 [49]			L**V**T**C**A**K**FAC	LD**K**QSDTQYV	**DII Dimer**
Z20 [53]			LVTCA**KFA**C	LD**K**QS**D**T**QY**V	**DII Dimer**
ZIKV-117 [51]			LVTCA**K**FAC	LDKQSDT**Q**YV	**DII Dimer**
A11 [49]			LVTCAKFAC		**DII Dimer**
1C11 [44]					**DIII**
Z3L1 [53]					**DI, DI-DII junction**
C10 [50]					**DIII/DII Dimer**

**Table 2:** Epitopes from ZIKV NS1 protein overlapping with critical binding residues of neutralizing monoclonal antibodies (mAbs). The mAbs are listed in the first column, and the epitopes with some degree of overlapping residues are listed in subsequent columns. Residues within epitopes that are critical for mAb interaction are shown in bold. The final column indicates the protein domain of each epitope.

	VREDYSLEC	KGPWHSEEL	CWYGMEIRPR	DOMAIN
3G2	VRE**DYSLE**C			**Wing domain**
z4B8	VRE**DYSLE**C			**Wing domain**
4F10		KGPW**H**SEEL		**β-ladder domain**
2E11			CWYGMEIRP**R**	**β-ladder domain**
14G5			CWYGMEIR**PR**	**β-ladder domain**

**Table 3:** Binding affinity values (ΔG in kcal/mol) obtained from molecular docking simulations of ZIKV epitopes with HLA class I. More negative values indicate stronger predicted binding.

HLA CLASS I (kcal/mol)	HLA-B35:01	HLA-B44:03	HLA-B51:01	HLA-B08:01	HLA-B40:01	HLA-A68:01	HLA-A11:01	HLA-B53:01	HLA-B07:02	HLA-A03:01	HLA-A02:01
LGLNTKNGS	−8.7	−8.3	−9.0	−8.0	−7.2	−7.8	−8.2	−9.0	−9.3	−7.5	−8.2
LRLKGVSYS	−8.5	−7.4	−8.3	−7.7	−7.4	−7.1	−7.9	−8.7	−9.2	−7.2	−7.3
LVTCAKFAC	−8.2	−8.4	−9.3	−9.2	−7.0	−8.9	−8.9	−9.8	−9.3	−7.8	−6.6
KGPWHSEEL	−8.7	−9.1	−9.1	−8.2	−8.2	−8.6	−7.3	−8.9	−8.7	−8.4	−7.8

**Table 4:** Binding affinity values (ΔG in kcal/mol) obtained from molecular docking simulations of ZIKV epitopes with HLA class II alleles. More negative values indicate stronger predicted binding.

HLA CLASS II (kcal/mol)	HLA DRB1 03:01	HLA DRB1 07:01	HLA DRB1 15:01	HLA DRB5 01:01
MCLALGGVL	−8.0	−7.5	−6.5	−7.8
NSPRAEATL	−7.5	−7.1	−8.9	−8.4
RLITANPVI	−9.3	−8.5	−8.0	−8.1
KWYGMEIRPR	−8.4	−8.4	−7.8	−8.7
VKGKEAVHS	−7.9	−7.2	−7.7	−7.6
VQLTVVVGS	−6.9	−8.5	−8.2	−8.1
VREDYSLEC	−7.0	−7.3	−9.1	−8.2
WRLKRAHLI	−8.1	−7.8	−8.3	−8.6

**Table 5:** Physicochemical, immunological, and safety properties of the EnvNS1 multiepitope construct. Values as per the table provided, including estimated half-life, solubility, pI, stability index, GRAVY (overall average hydropathy), aliphatic index, antigenicity, toxicity, and allergenicity.

Multi-Epitope Analyses	EnvNS1
Estimated Half-life: Mammary reticulocytes (in vitro): 30 h Yeast (in vivo): 20 h *Escherichia coli* (in vivo): 10 h	30 h 20 h 10 h
Solubility (>0.45)	0.566 (Soluble)
Theoretical pI (>7 basic)	8.74 (Basic)
Instability Index (<40)	30.39 (Stable)
Average Hydropathy Score (>0.0 hydrophilic)	−0.235 (Hydrophilic)
Aliphatic Index (>70)	76.91 (Thermostable)
Antigenicity (>0.4)	0.4486 (Probable antigen)
Toxicity (>0.0)	0.3 (Non-toxic)
Allergenicity	Likely non-allergen

**Table 6:** Alignment of the synthetic EnvNS1 antigen against the human proteome and clinically important flaviviruses. Results show the organism and its respective TaxID, description of the main alignment hit, alignment score, query coverage, probability of the alignment occurring by chance (E-value), identity and accession length of the aligned sequence.

Organism (TaxID)	Description	Max Score	Query Cover	E-Value	Percent Identity (%)	Acc. Len
Dengue virus (12637)	NS1 protein [dengue virus type 4]	28.1	4%	0.018	81.82	NP_740318.1
West Nile virus (11082)	polyprotein [West Nile virus]	37.0	14%	3 × 10^−5^	40.54	YP_001527877.1
Japanese encephalitis virus (11072)	envelope protein	33.5	25%	1 × 10^−4^	29.89	NP_775666.1
Yellow fever virus (11089)	No significant similarity found	-	-	-	-	-
Homo sapiens (9606)	No significant similarity found	-	-	-	-	-

**Table 7:** Population coverage of HLA alleles and the allele set identified as binding to the EnvNS1 epitopes.

Epitope	World	South America	Brazil
LGLNTKNGS	61.74	40.86	58.69
LVTCALKFAC	37.38	24.71	34.40
NSPRAEATL	34.78	14.26	13.93
RLITANPVI	34.44	13.72	14.49
WRLKRAHLI	34.44	13.72	14.49
KGPWHSEEL	28.80	13.80	23.20
LRLKGVSYS	20.79	8.38	16.96
KWYGMEIRPR	17.84	8.76	11.24
MCLALGGLVL	17.84	8.76	11.24
VREDYSLEC	17.84	8.76	11.24
Epitope set	87.28	60.59	75.83

**Table 8:** Th2/Th1 ratios for plasma antibodies. IgG isotypes were identified in the plasma of immunized mice at the conclusion of the experimental timeline.

IgG1/IgG2a			IgG2b/IgG2a		
pVAX1	EnvNS1	ssEnvNS1	pVAX1	EnvNS1	ssEnvNS1
1.0405	0.8083	0.7482	1.1572	0.9558	0.8879

## Data Availability

The data presented in this study are available upon request from the corresponding author.

## Supplementary Materials

The following supporting information can be downloaded at https://www.mdpi.com/article/10.3390/vaccines14010031/s1. Figure S1: Molecular docking analysis; Figure S2: Molecular interactions between the epitope complexes and the residues present in the binding pockets of class I and class II HLAs; Figure S3: Variations in the stability parameters of the complexes of HLA class I throughout the simulation.; Figure S4: Variations in the stability parameters of the complexes of HLA class II throughout the simulation; Figure S5: Integrated heatmap of structural stability scores obtained from molecular dynamics; Figure S6: Ramachandran plots of psi and phi angle distributions in the predicted antigen structure; Figure S7: Structural mapping of epitopes and linker conformations in predicted antigen; Figure S8: Confirmation of cloning of vaccine candidates; Figure S9: Gating strategy for immunophenotyping analyses. Table S1: Prediction parameters of antigenicity and MHC class I immunogenicity of the identified MHC binding epitopes; Table S2: Zika virus strain sequences used for consensus epitope identification; Table S3: Mean and standard deviation of the weight of groups of mice throughout the immunization experiment; Table S4: Hematological parameters of immunized mice and control group (mean and standard deviation per group); Table S5: Biochemical parameters of immunized mice and control group (mean and standard deviation per group).

## Abbreviations

The following abbreviations are used in this manuscript:

Å	Angstrom
ADE	Antibody-Dependent Enhancement
BLASTp	Basic Local Alignment Search Tool (protein)
C	Capsid protein
CD4	Cluster of Differentiation 4
CD8	Cluster of Differentiation 8
CENAPAD-SP	Centro Nacional de Processamento de Alto Desempenho em São Paulo
CHARMM	Chemistry at Harvard Macromolecular Mechanics
CTL	Cytotoxic T Lymphocytes
CZS	Congenital Zika Syndrome
DENV	Dengue Virus
DI	Domain I (of the E protein)
DII	Domain II (of the E protein)
DIII	Domain III (of the E protein)
DMEM	Dulbecco’s Modified Eagle Medium
DNA	Deoxyribonucleic Acid
E	Envelope protein
*E. coli*	*Escherichia coli*
ER	Endoplasmic Reticulum
EnvNS1-Epi	Envelope and NS1 multi-epitope construct
FITC	Fluorescein Isothiocyanate
ΔG	Free binding energy
GDT	Global Distance Test
GRAVY	Grand Average of Hydropathy
HEK 293T	Human Embryonic Kidney 293T cells
HIS-tag	Histidine tag
HLA	Human Leukocyte Antigen
IFN-γ	Interferon gamma
Ig	Immunoglobulin
IgG	Immunoglobulin G
IgG1	Immunoglobulin G subclass 1
IgG2	Immunoglobulin G subclass 2
IgM	Immunoglobulin M
IEDB	Immune Epitope Database
IL	Interleukin
IL-2	Interleukin-2
IL-4	Interleukin-4
IL-10	Interleukin-10
IL-12	Interleukin-12
JEV	Japanese Encephalitis Virus
K	Kelvin
kcal/mol	kilocalories per mole
mAb	Monoclonal Antibody
mAbs	Monoclonal Antibodies
MD	Molecular Dynamics
MHC	Major Histocompatibility Complex
NAMD	Nanoscale Molecular Dynamics
ns	nanoseconds
NS1	Nonstructural Protein 1
PDB	Protein Data Bank
pH	Potential of Hydrogen
pI	Isoelectric Point
PROPRED	PROtein PREDiction tool
pTM	predicted Template Modeling
pVAX_EnvNS1	pVAX1 plasmid containing the EnvNS1 gene
pVAX_ssEnvNS1	pVAX1 plasmid containing the ssPGIP-EnvNS1 gene
PyRx	Python Prescription
RMSD	Root Mean Square Deviation
RMSF	Root Mean Square Fluctuation
ssPGIP	Signal peptide from the Polygalacturonase-Inhibiting Protein
Tfh	T follicular helper
TGF-β	Transforming Growth Factor beta
Th1	T helper 1
Th2	T helper 2
Th17	T helper 17
TNF	Tumor Necrosis Factor
VERO	Vero cell line (from African green monkey kidney)
VMD	Visual Molecular Dynamics
WNV	West Nile Virus
YFV	Yellow Fever Virus
ZIKV	Zika Virus
