# Supplementary material for: Development and Immunogenicity Assessment of a Multi-Epitope Antigen Against Zika Virus: An In Silico and In Vivo Approach
Source: Vaccines (Basel). 2025 Dec 26;14(1):31. doi: 10.3390/vaccines14010031 (PMC12846674; doi:10.3390/vaccines14010031)
Supplement: Supplementary file 1 [file vaccines-14-00031-s001.zip › vaccines-4066256-supplementary.pdf]

Supplementary Materials

HLA	PDB Code	HLA class I binding complex - epitopes			
		KGPWHSEEL	LGLNTKNGS	LRLKGVSYS	LVTCAKFAC
HLA-A*11:01	7m8t	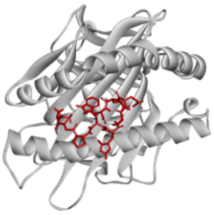	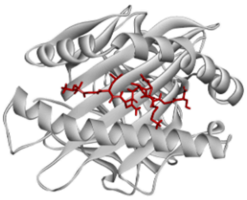	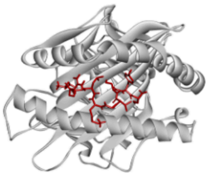	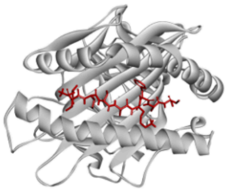
HLA-B*53:01	7r80	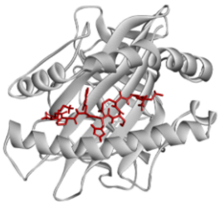	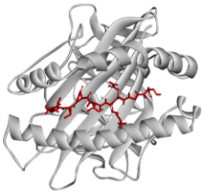	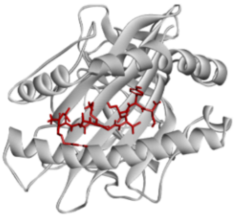	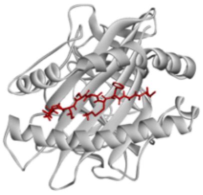
HLA-B*07:02	7s79	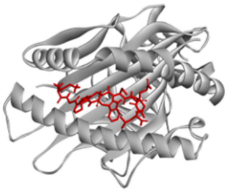	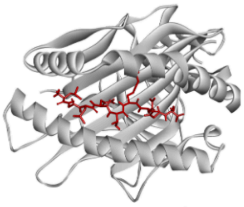	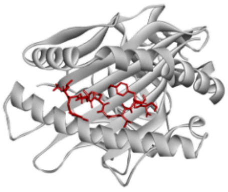	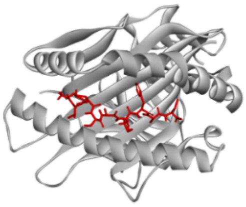
HLA-A*03:01	7uc5	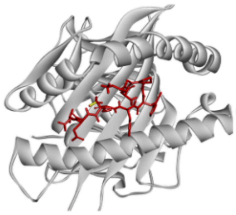	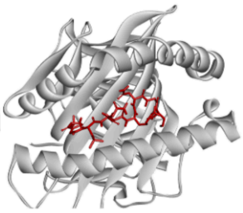	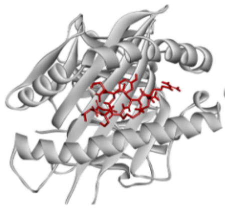	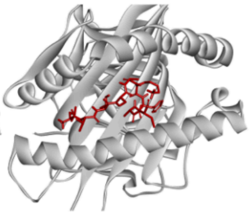
HLA-A*02:01	7um2	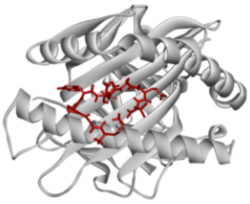	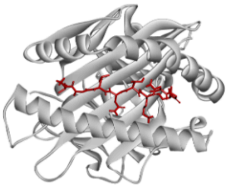	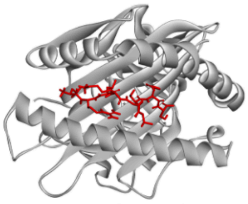	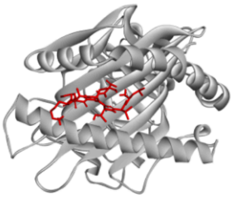
HLA-B*58:01	7x00	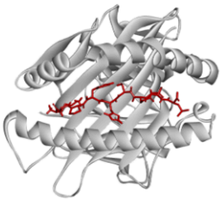	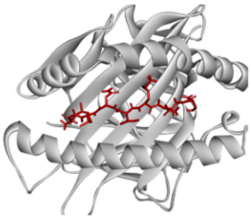	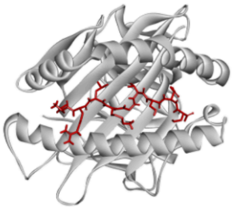	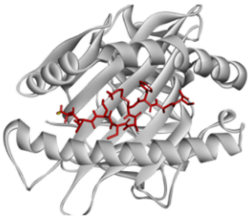

HLA	PDB Code	HLA class I binding complex - epitopes			
		KGPWHSEEL	LGLNTKNGS	LRLKGVSYS	LVTCAKFAC
HLA-B*35:01	1XH3	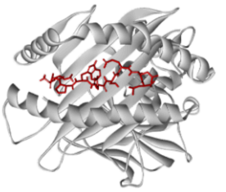	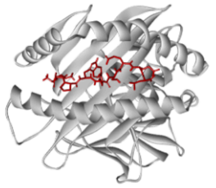	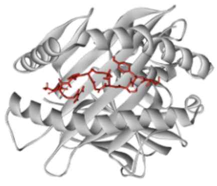	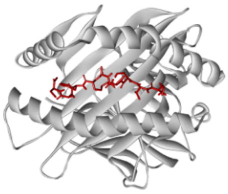
HLA-B*44:03	4jqx	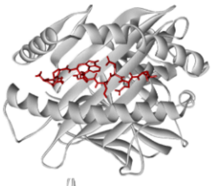	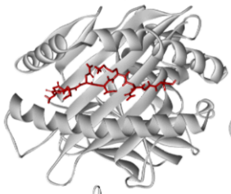	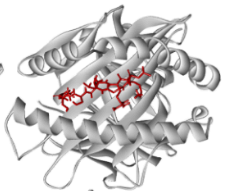	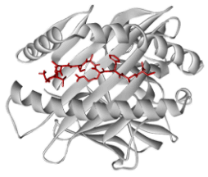
HLA-B*51:01	4mji	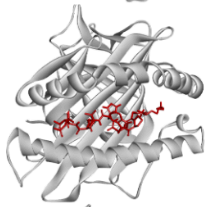	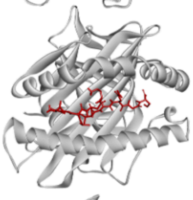	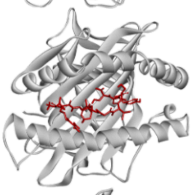	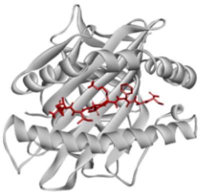
HLA-B*08:01	4qrs	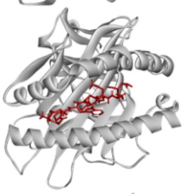	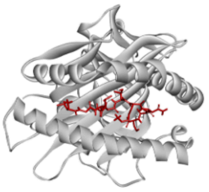	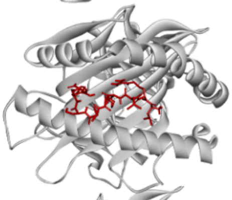	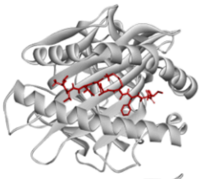
HLA-B*40:01	6iex	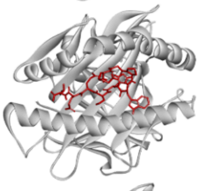	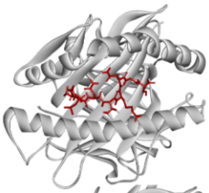	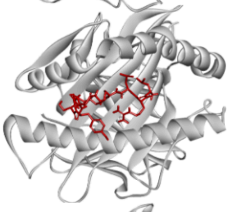	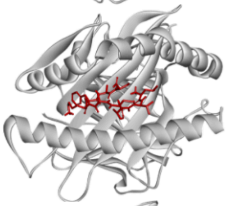
HLA-A*68:01	6pbh	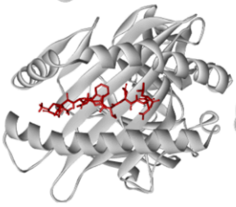	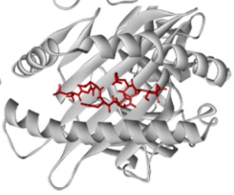	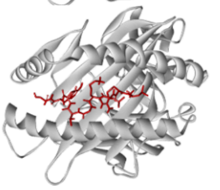	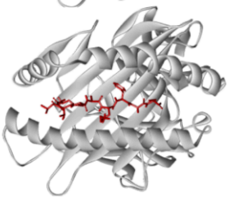

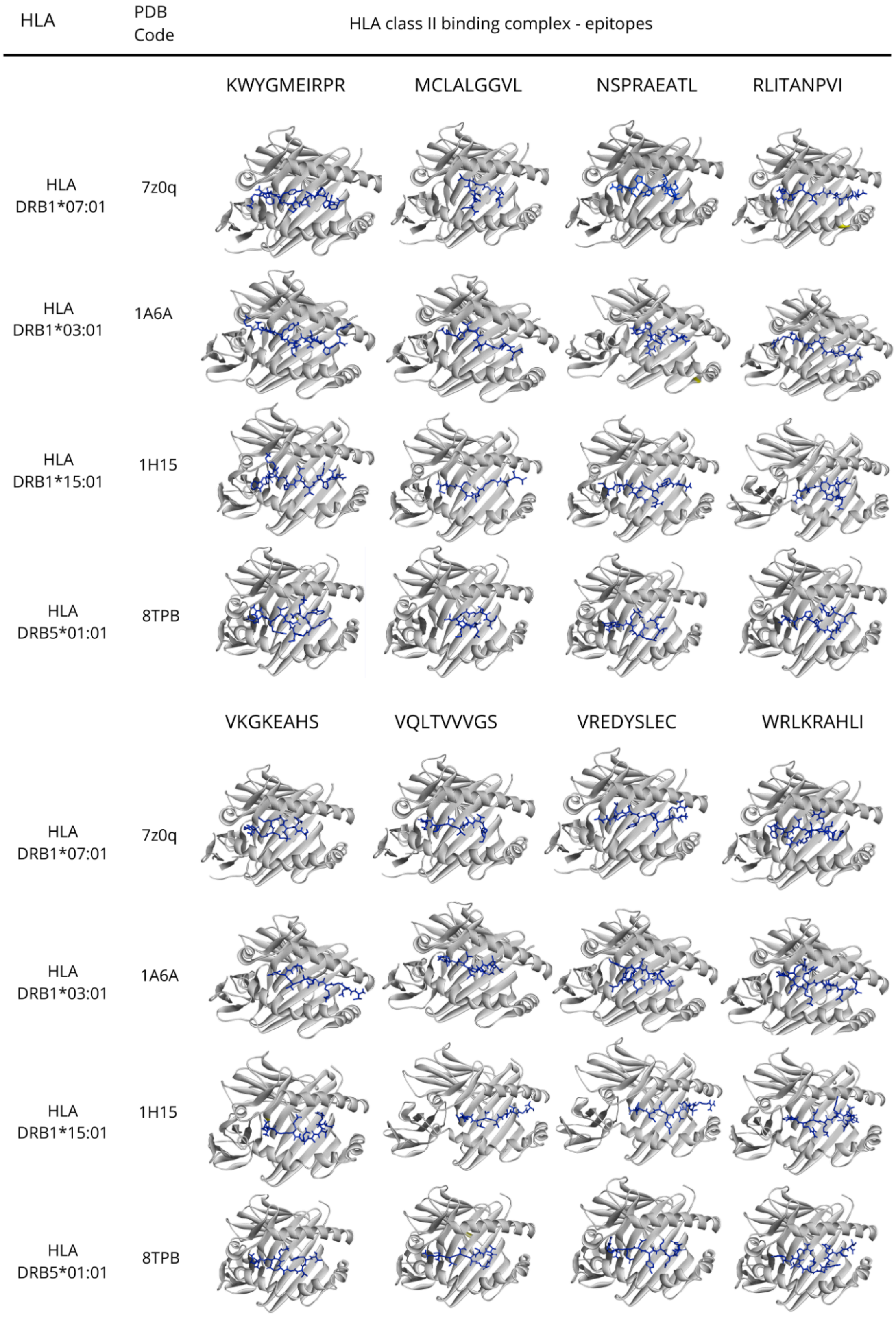

Figure S1: Molecular docking analysis. The molecular docking analysis of the complexes shows the binding conformation of the epitopes within the peptide-binding groove of HLA class I receptors (in red) and HLA class II receptors (in blue). Epitopes are represented in the columns and the corresponding HLAs in the rows.

HLA	Molecular interactions of HLA class I binding complex - epitopes			
	KGPWHSEEL	LGLNTKNGS	LRLKGVSYS	LVTCAKFAC
HLA-B*35:01	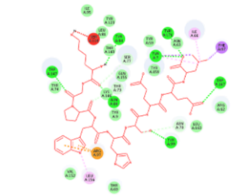	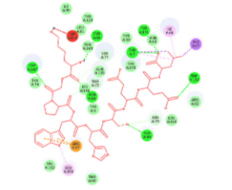	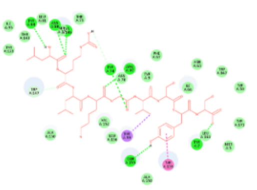	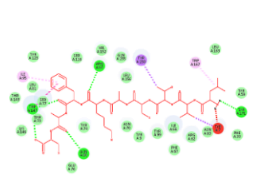
HLA-B*44:03	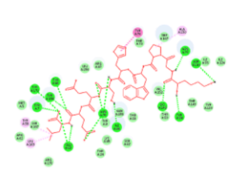	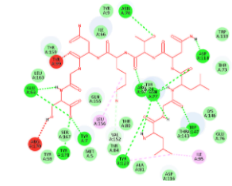	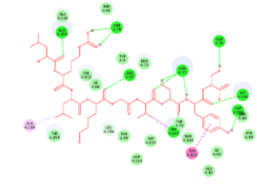	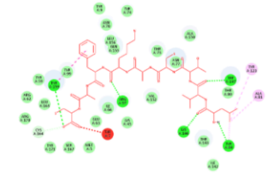
HLA-B*51:01	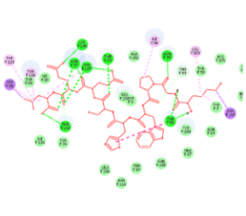	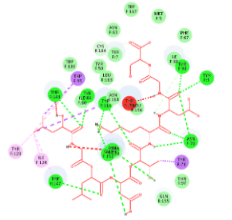	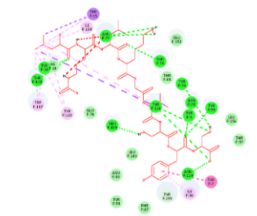	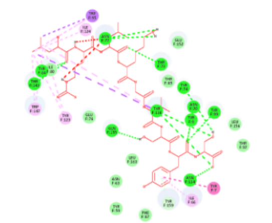
HLA-B*08:01	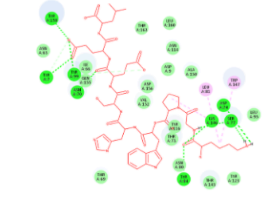	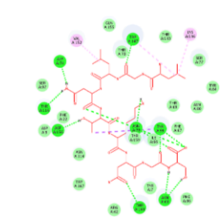	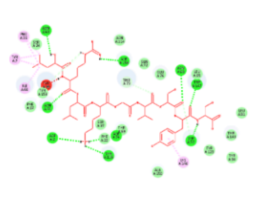	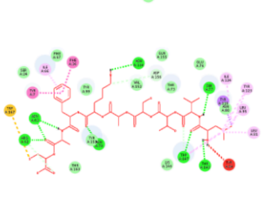
HLA-B*40:01	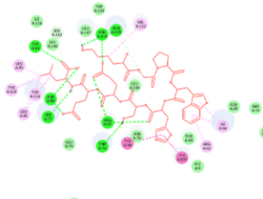	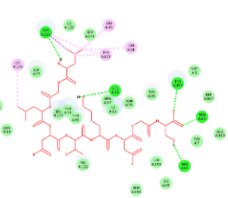	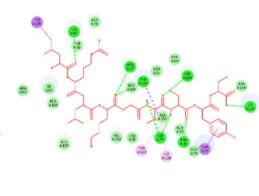	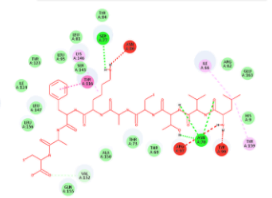
HLA-A*68:01	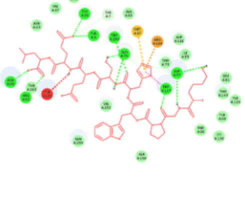	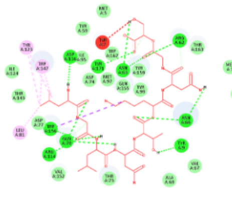	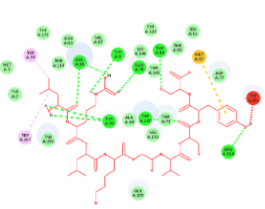	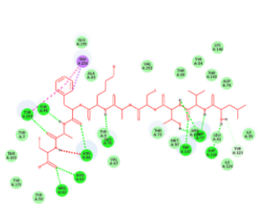

HLA	Molecular interactions of HLA class I binding complex - epitopes			
	KGPWHSEEL	LGLNTKNGS	LRLKGVSYS	LVTCAKFAC
HLA-A*11:01				
HLA-B*53:01				
HLA-B*07:02				
HLA-A*03:01				
HLA-A*02:01				
HLA-B*58:01				

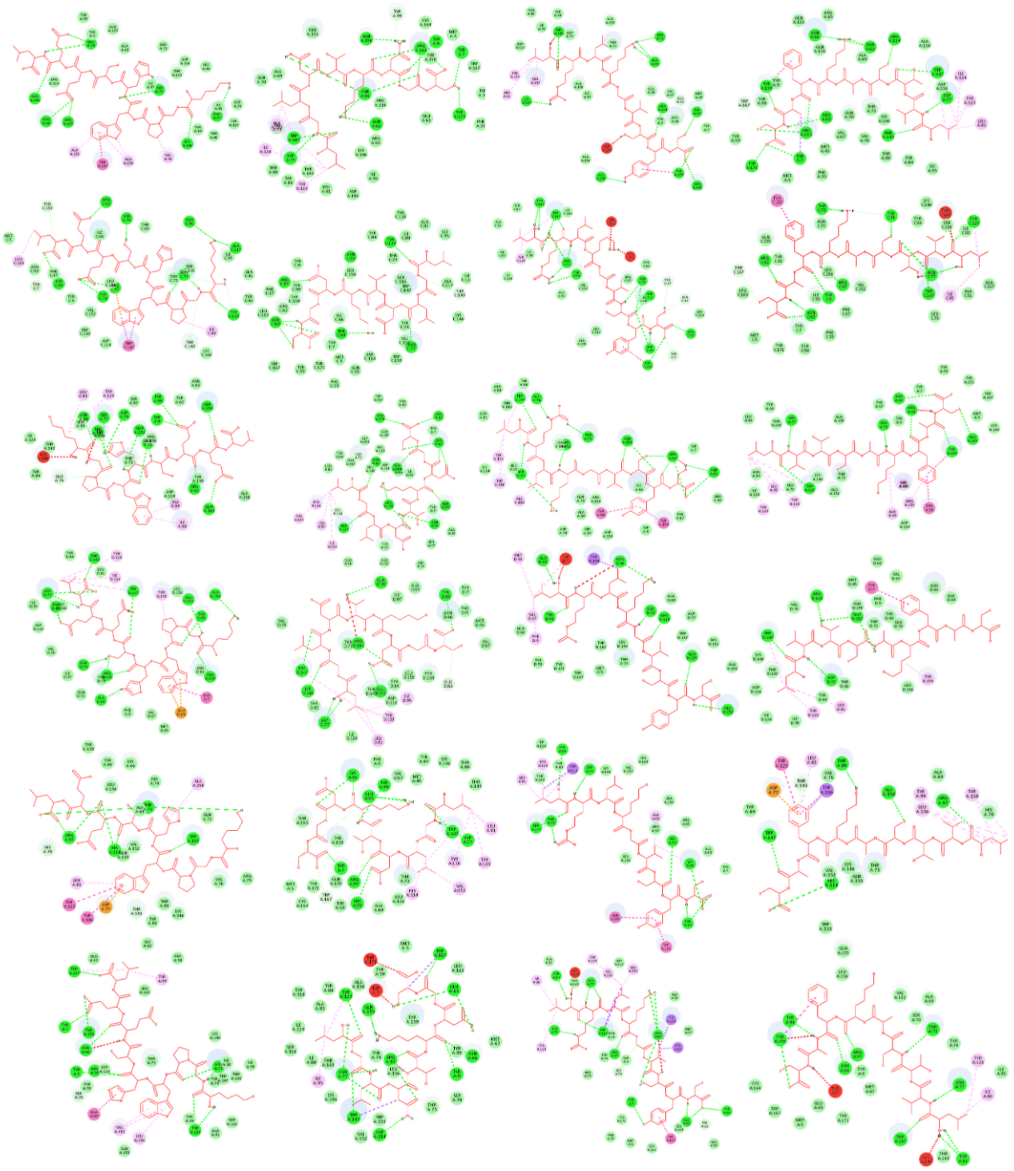

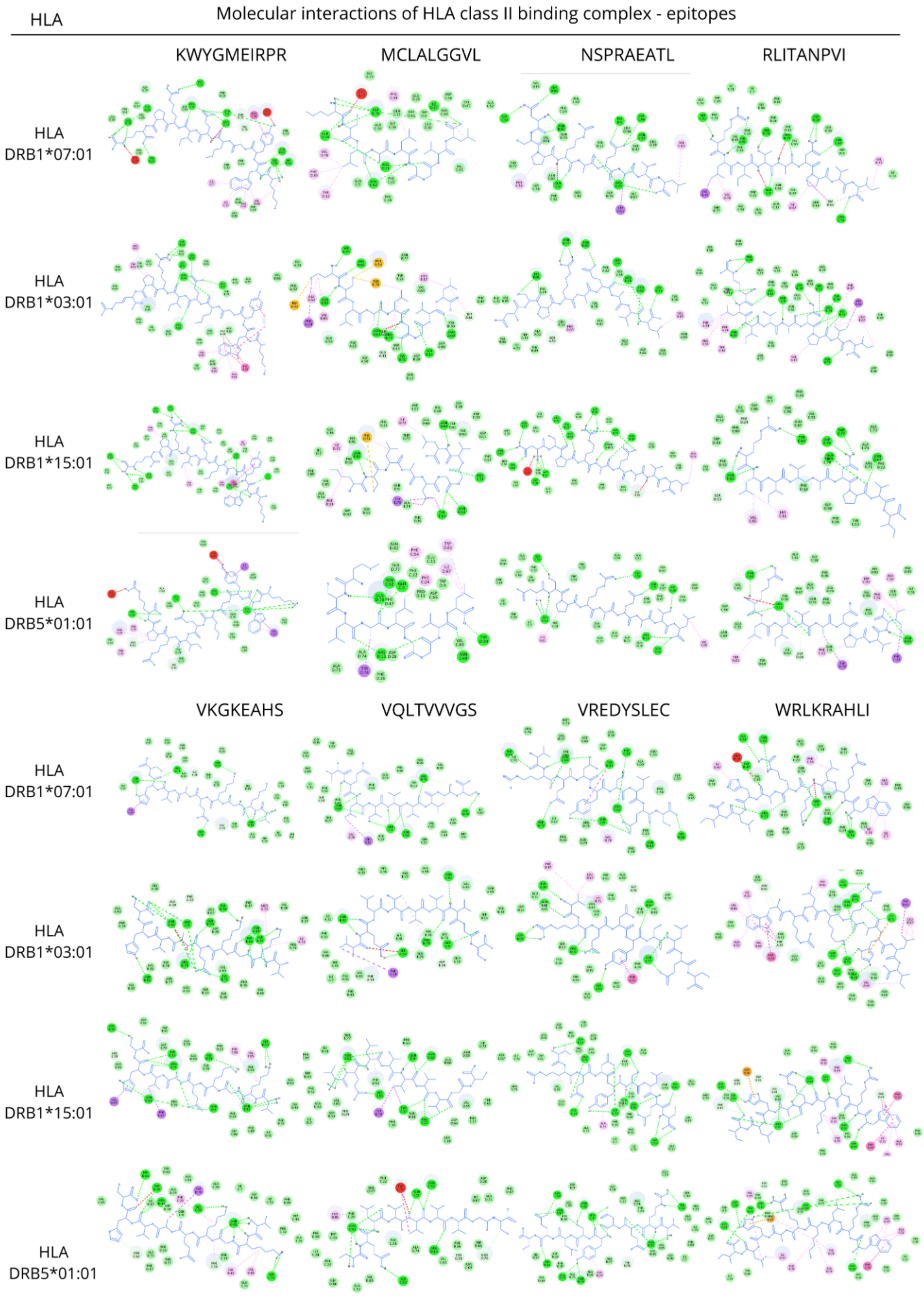

Figure S2: Molecular interactions between the epitope complexes and the residues present in the binding pockets of class I and class II HLAs. The schematic representation of the interactions follows the color scheme of Figure 3.

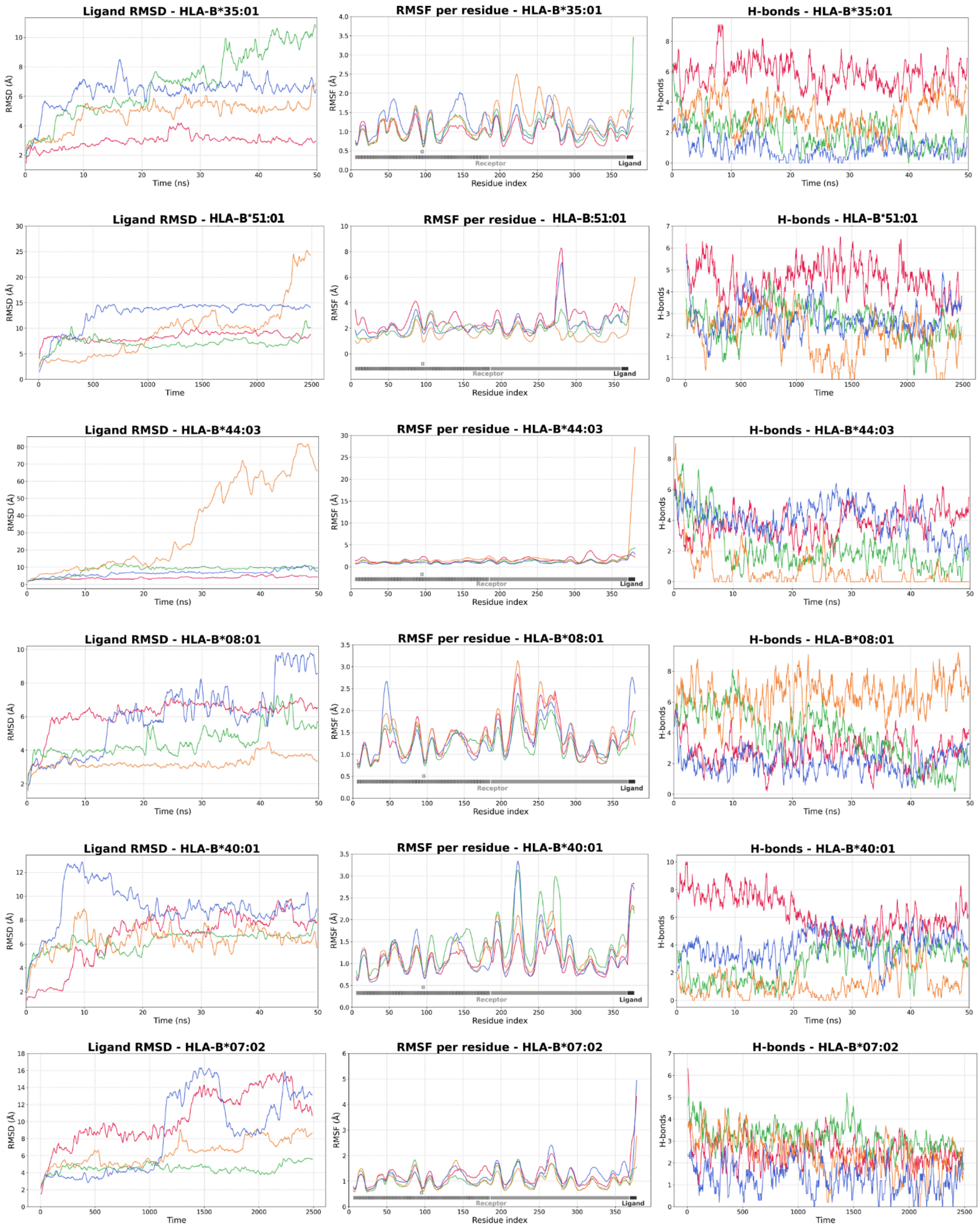
Ligand RMSD - HLA-B*35:01
RMSD (Å)
Time (ns)
RMSF per residue - HLA-B*35:01
RMSF (Å)
Residue index
Receptor
Ligand
H-bonds - HLA-B*35:01
H-bonds
Time (ns)
Ligand RMSD - HLA-B*51:01
RMSD (Å)
Time
RMSF per residue - HLA-B:51:01
RMSF (Å)
Residue index
Receptor
Ligand
H-bonds - HLA-B*51:01
H-bonds
Time
Ligand RMSD - HLA-B*44:03
RMSD (Å)
Time (ns)
RMSF per residue - HLA-B*44:03
RMSF (Å)
Residue index
Receptor
Ligand
H-bonds - HLA-B*44:03
H-bonds
Time (ns)
Ligand RMSD - HLA-B*08:01
RMSD (Å)
Time (ns)
RMSF per residue - HLA-B*08:01
RMSF (Å)
Residue index
Receptor
Ligand
H-bonds - HLA-B*08:01
H-bonds
Time (ns)
Ligand RMSD - HLA-B*40:01
RMSD (Å)
Time (ns)
RMSF per residue - HLA-B*40:01
RMSF (Å)
Residue index
Receptor
Ligand
H-bonds - HLA-B*40:01
H-bonds
Time (ns)
Ligand RMSD - HLA-B*07:02
RMSD (Å)
Time
RMSF per residue - HLA-B*07:02
RMSF (Å)
Residue index
Receptor
Ligand
H-bonds - HLA-B*07:02
H-bonds
Time

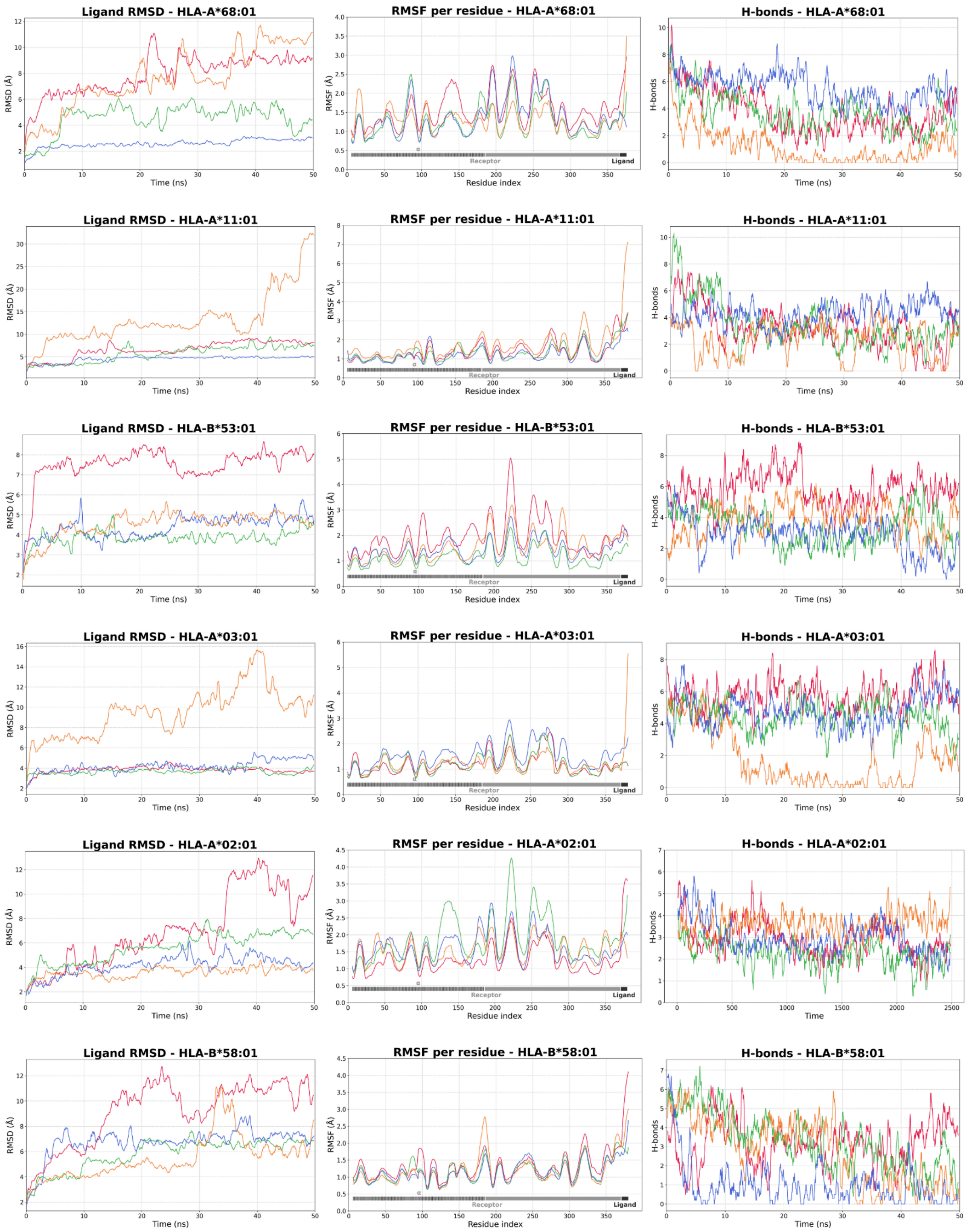

Figure S3: Variations in the stability parameters of the complexes of HLA class I throughout the simulation. RMSD (Å) of the epitopes (colored) within the binding groove; RMSF of the complex residues, with the bar representing the arrangement of residues in the complex structure. The receptor region is shown in gray, the binding site region and its designated chain are represented with pattern fill, and the ligand is shown in black; Finally, the formation of hydrogen bonds between the ligand and the binding site of each corresponding HLA is presented.

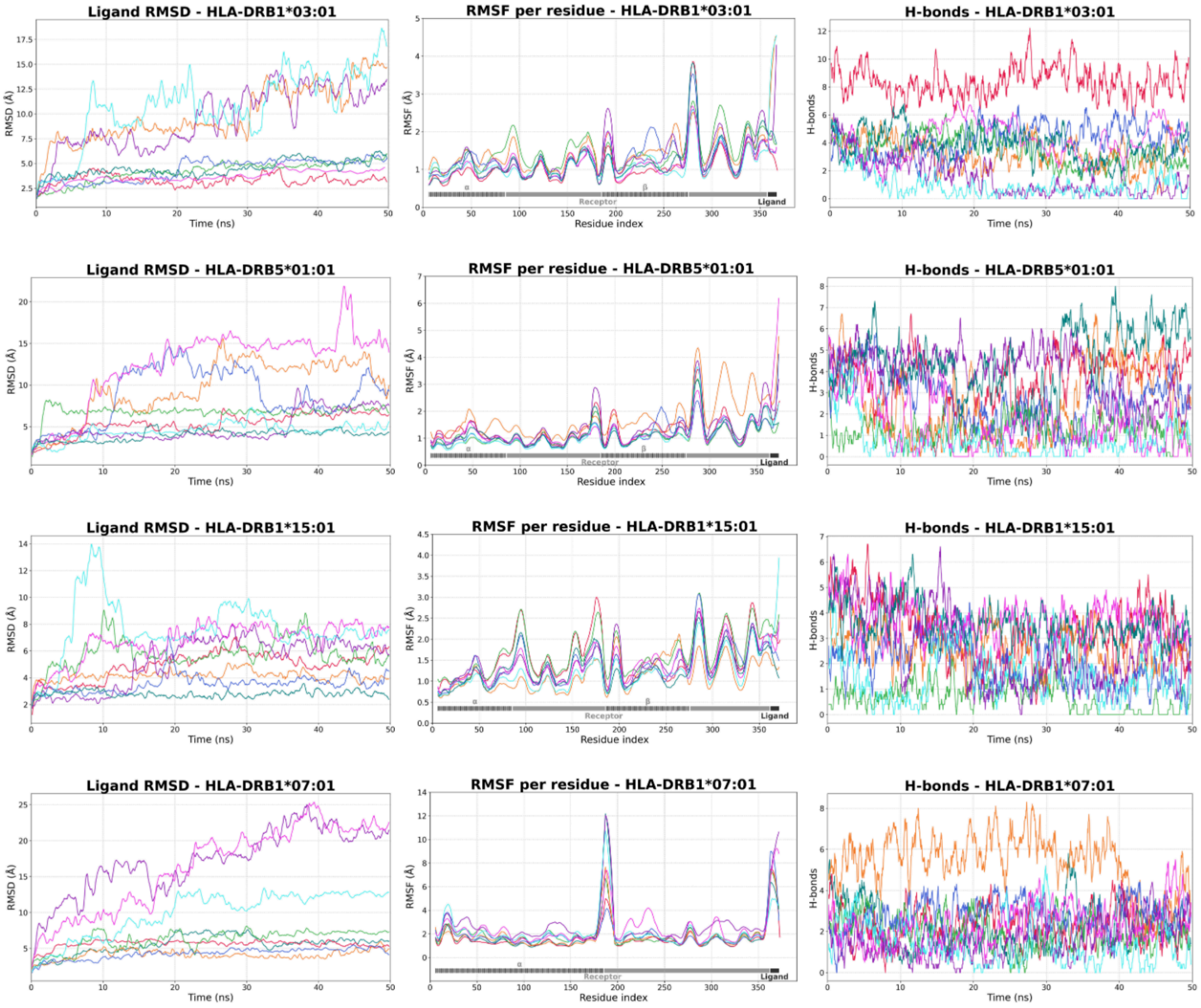

Figure S4: Variations in the stability parameters of the complexes of HLA class II throughout the simulation. RMSD (Å) of the epitopes (colored) within the binding groove; RMSF of the complex residues, with the bar representing the arrangement of residues in the complex structure. The receptor region is shown in gray, the binding site region and its designated chain are represented with pattern fill, and the ligand is shown in black; Finally, the formation of hydrogen bonds between the ligand and the binding site of each corresponding HLA is presented.

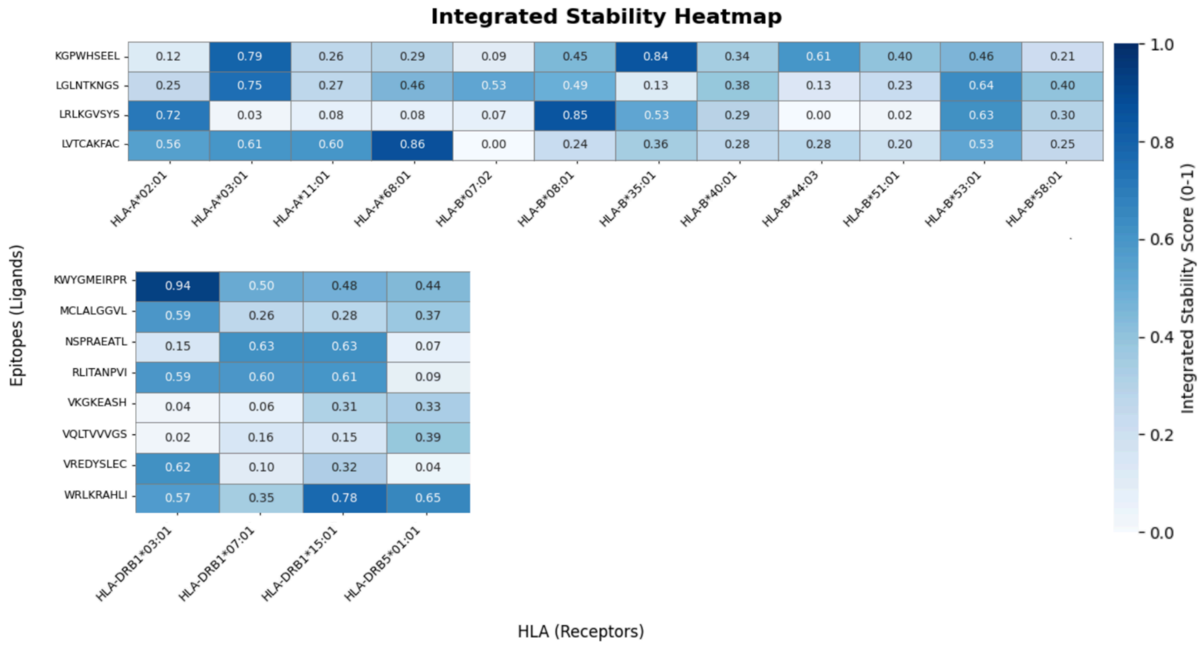

Figure S5: Integrated heatmap of structural stability scores obtained from molecular dynamics. Colorations closer to white indicate lower stability, with low formation of hydrogen bonds between the epitope and the receptor. Darker shades of blue indicate higher structural stability, with better results in RMSD, RMSF, and number of hydrogen bond parameters.

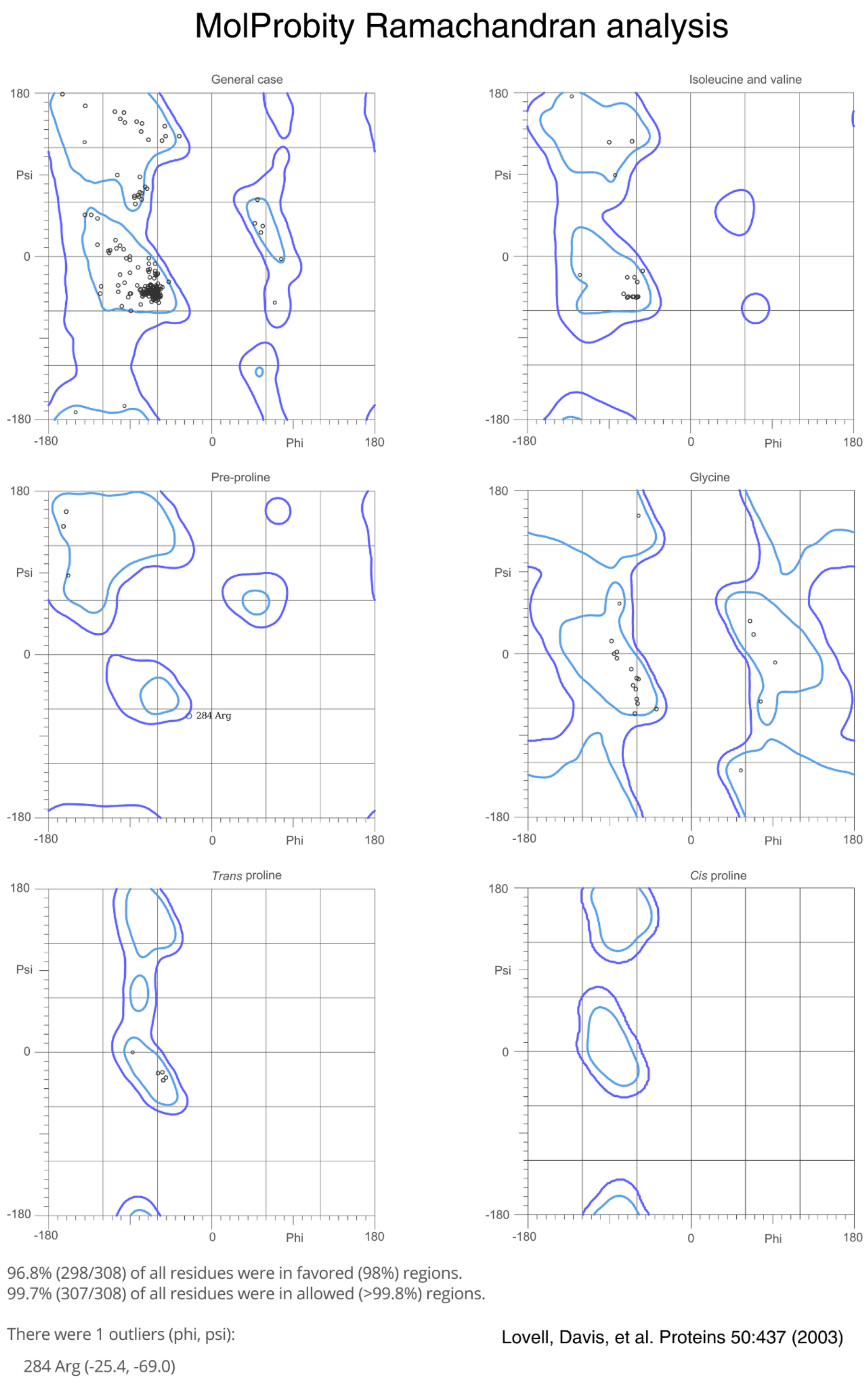

Figure S6: Ramachandran plots of psi and phi angle distributions in the predicted antigen structure. General case shows common allowed angles; residue-specific plots (Isoleucine, Valine, Pre-proline, Glycine, Trans-proline, Cis-proline) highlight structural constraints.

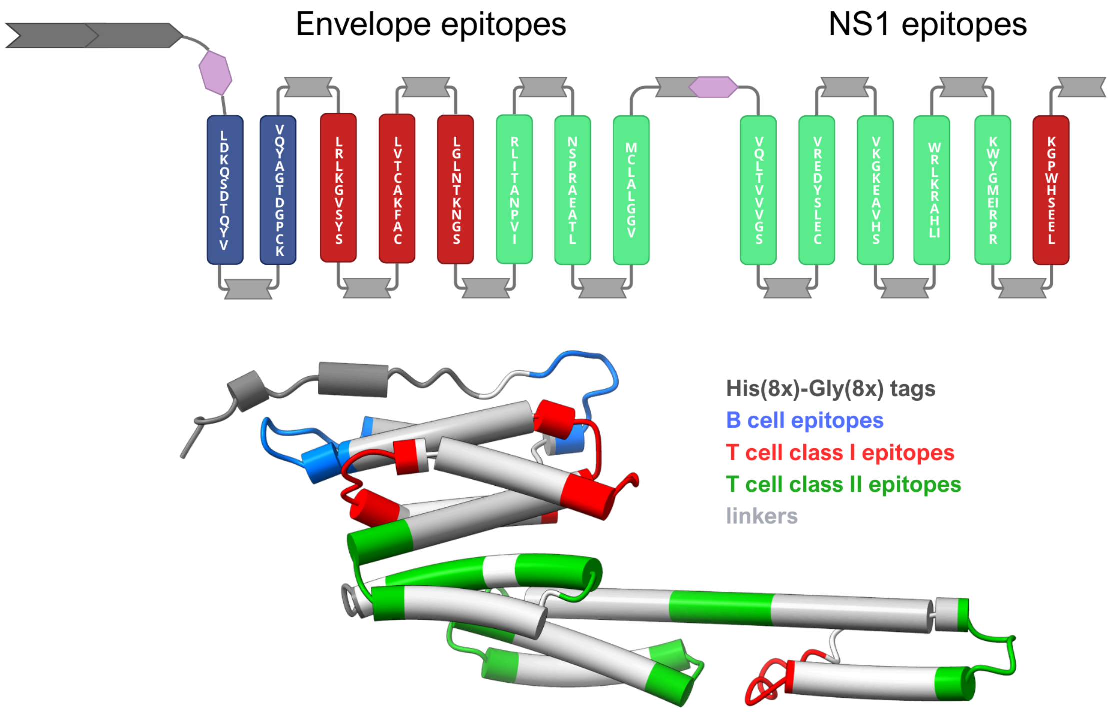

Figure S7: Structural mapping of epitopes and linker conformations in predicted antigen. Dark grey: His(8x) and Gly(8x) tags; light grey: concatenated linkers; blue: B-cell epitopes; red: MHC class I epitopes; green: MHC class II epitopes.

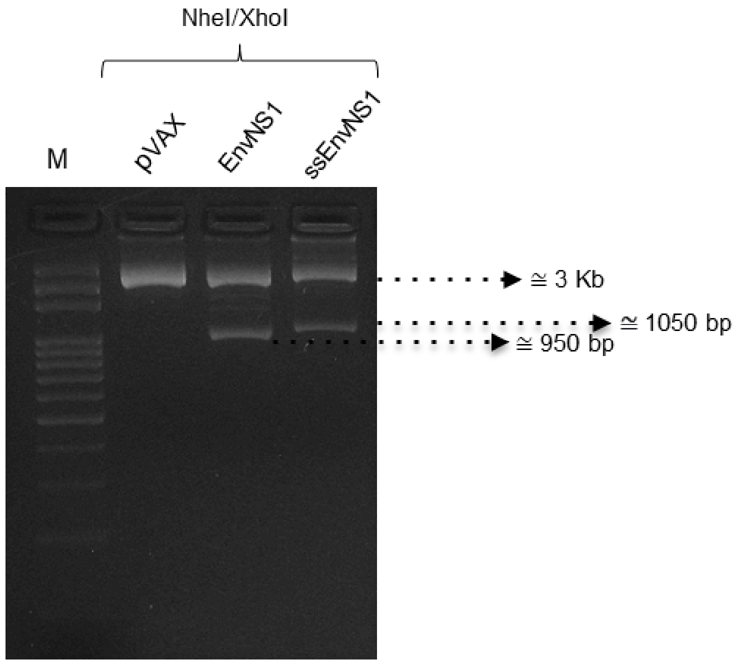

Figure S8: Confirmation of cloning of vaccine candidates. Enzymatic digestions of the empty vector pVAX1 (3 kb) and the recombinants pVAX1_EnvNS1 (3948 bp) and pVAX1_ssEnvNS1 (4051 bp) with NheI and XhoI enzymes were done to verify cloning of inserts. M: Molecular weight marker 100 bp DNA Ladder Ready to Load (Solis BioDyne).

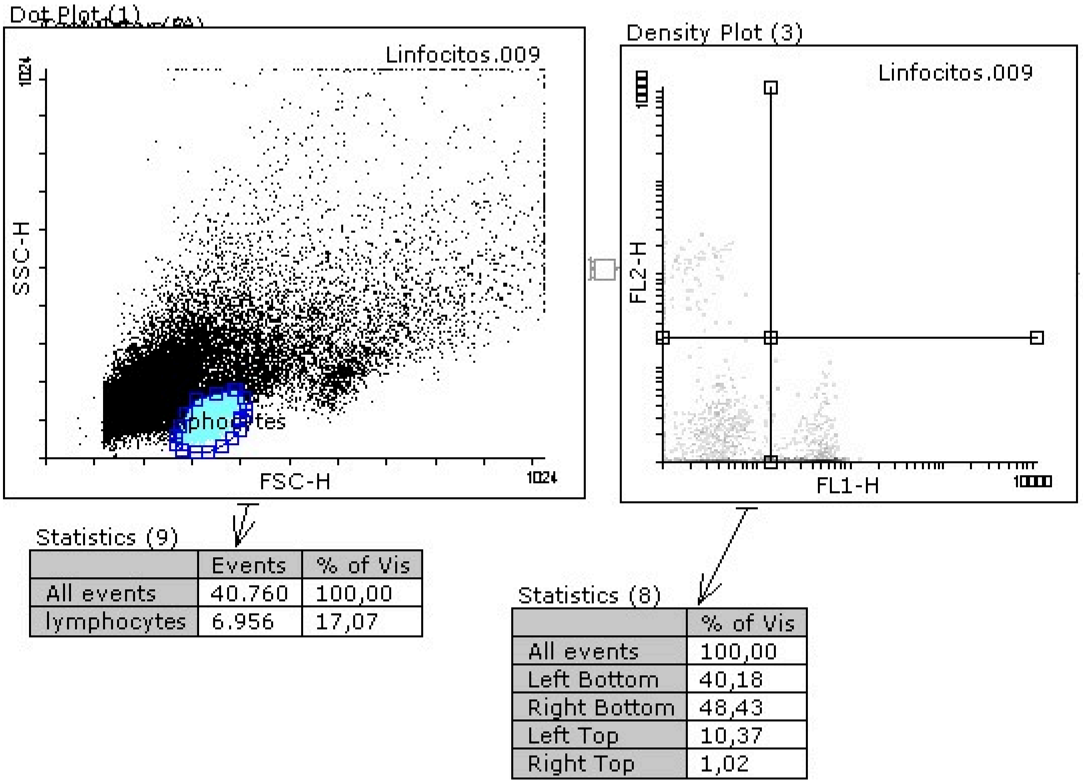

Figure S9: Gating strategy for immunophenotyping analyses.

Epitope	Antigenicity score (>0.5)	Class I immunogenicity score
KGPWHSEEL	0.82	0.24
NSPRAEATL	0.79	0.23
MCLALGGVL	1.36	0.11
LRLKGVSYS	1.53	-
LGLNTKNGS	2.06	-
LVTCAKFAC	1.23	-
VREDYSLEC	1.69	-
VKGKEAVHS	0.92	-
VQLTVVVGS	0.79	-
WRLKRAHLI	1.02	-

WYGMEIRPR	1.01	-
RLITANPVI	-	-
LDKQSDTQYV	1.04	-
EVQYAGTDGPCK	1.03	-

Table S1: Prediction parameters of antigenicity and MHC class I immunogenicity of the identified MHC binding epitopes.

Accession number	Country/Region
ANW07475.1	Malaysia / Southeast Asia
ACD75819.1	Micronesia / Oceania
AMR39834.1	Cambodia / Southeast Asia
AMC3912.1	Guatemala / Central America
KU509998.1	Haiti / North America
ALU33341.1	Brazil / South America
AHL37808.1	Canada / North America
AMC33116.1	Martinique / Caribe
ALX35661.1	Suriname / South America
ALX35660.1	Suriname / South America
ALX35659.1	Suriname / South America
AMC13911.1	Puerto Rico / Central America
AMA12084.1	Brazil / South America
AMA12085.1	Brazil / South America
AHZ13508.1	French Polynesia / Oceania
AHL43503.1	Senegal / East Africa
AHL43502.1	Senegal / East Africa
AEN75265.1	Nigeria / East Africa
AHL43501.1	Senegal / East Africa
AEN75266.1	Senegal / East Africa
AHL43505.1	Senegal / East Africa
AHL43504.1	Senegal / East Africa
YP_002790881.1	Senegal, Uganda / East Africa
BAP47441.1	Uganda / East Africa

AEN75263.1	Uganda / East Africa
ABI54475.1	Uganda / East Africa
AHL43500.1	Senegal, Central African Republic / Central Africa
AHF49784.1	Central African Republic / Central Africa
AHF49785.1	Central African Republic / Central Africa
AHF49783.1	Central African Republic / Central Africa
AMD61711	Philippines / Southeast Asia
AMD61710	Thailand / Southeast Asia
AMO03410	China / Northeast Asia
AMH87239	Brazil / South America
AMA12087	Brazil / South America
AMQ34004	Mexico: Chiapas / North America
AMQ34003	Mexico: Chiapas / North America
AMM39805	China / Northeast Asia
AMK79468	China / Northeast Asia
AMN14620	Italy: Padua / Europe
AMN14619	Italy: Padua / Europe
AMK49164	Brazil / South America
AMK49165	Brazil / South America
AML82110	China / Northeast Asia
AMK79469	China / Northeast Asia
AMD16557	Brazil / South America
AMB18850	Brazil: Rio Grande do Norte, Natal / South America
AMC13913	Guatemala / Central America
AMC13912	Guatemala / Central America
AMB37295	Haiti / North America
AAV34151	Uganda / East Africa
AMM39806	China / Northeast Asia
AMM39804	Colombia: Barranquilla / South America
AMK02027	Uganda / East Africa

Table S2: Zika virus strain sequences used for consensus epitope identification.

Groups	Weight (g)		
	Day 1	Day 7	Day 21
**pVAX1**	21.12 ± 0.53	21.99 ± 0,57	23.17 ± 0.53
**pVAX_EnvNS1**	19.36 ± 1.99	20.31 ± 1.93	21.35 ± 2.14
**pVAX_ssEnvNS1**	21.02 ± 1.52	22.04 ± 1.28	22.79 ± 2.40

Table S3: Mean and standard deviation of the weight of groups of mice throughout the immunization experiment.

Parameters	pVAX1	EnvNS1	ssEnvNS1	Mean ± SD
Red blood cells ($10^6/mm^3$)	4.58 ± 0.28	4.94 ± 0.61	4.61 ± 0.24	4.71 ± 0.16
Hemoglobin (g/dL)	13.5 ± 0.91	14.62 ± 2.06	13.62 ± 0.84	13.91 ± 0.5
Hematocrit (%)	40.72 ± 2.72	44.00 ± 6.22	41.00 ± 2.54	41.91 ± 1.48
VCM (fL)	89.24 ± 1.83	90.76 ± 3.46	90.32 ± 2.70	90.11 ± 0.64
CHCM (%)	33.00 ± 0	33.12 ± 0.14	32.97 ± 0.10	33.02 ± 0.06
Total leukocytes ($10^3/mm^3$)	9.02 ± 0.41	10 ± 0.42	9.07 ± 0.74	9.36 ± 0.45
Neutrophils (%)	40.2 ± 4.90	34.00 ± 5.21	39.25 ± 4.02	37.82 ± 2.73
Lymphocytes (%)	56.8 ± 5.41	62.8 ± 4.79	58.25 ± 3.69	59.28 ± 2.56
Eosinophils (%)	1.2 ± 0.40	1.6 ± 0.80	1.25 ± 0.43	1.35 ± 0.18

Monocytes (%)	1.8 ± 0.97	1.6 ± 0.80	1.25 ± 0.43	1.55 ± 0.23
Platelets ($10^3/mm^3$)	417.4 ± 62.92	412.4 ± 67.00	433.5 ± 33.26	421.10 ± 9.00

Table S4: Hematological parameters of immunized mice and control group (mean and standard deviation per group).

**Parameters**	**pVAX1**	**EnvNS1**	**ssEnvNS1**	**Mean ± SD**
Glucose	74.28 ± 5.68	77.12 ± 3.72	74.97 ± 7.13	75.46 ± 1.21
Urea (mg/dL)	64.38 ± 1.33	69.58 ± 2.73	67.45 ± 3.66	67.14 ± 2.13
Creatinine (mg/dL)	0.56 ± 0.08	0.61 ± 0.06	0.53 ± 0.04	0.57 ± 0.03
AST (UI/L)	130.32 ± 4.52	131.22 ± 3.22	130.6 ± 2.16	130.71 ± 0.38
ALT (UI/L)	152.44 ± 5.86	150.5 ± 5.50	147.85 ± 3.55	150.26 ± 1.88
FAL (UI/L)	223.84 ± 5.29	220.72 ± 3.43	225.4 ± 4.64	223.32 ± 1.95

Table S5: Biochemical parameters of immunized mice and control group (mean and standard deviation per group).
